# Lipid Profiles From Fresh Biofilms Along a Temperature Gradient on a Hydrothermal Stream at El Tatio (Chilean Andes), as a Proxy for the Interpretation of Past and Present Biomarkers Beyond Earth

**DOI:** 10.3389/fmicb.2022.811904

**Published:** 2022-06-27

**Authors:** Valentine Megevand, Daniel Carrizo, María Ángeles Lezcano, Mercedes Moreno-Paz, Nathalie A. Cabrol, Víctor Parro, Laura Sánchez-García

**Affiliations:** ^1^Centro de Astrobiología (CAB), INTA-CSIC, Madrid, Spain; ^2^Department of Earth Sciences, Ecole Normale Supérieure de Lyon, Université Claude Bernard Lyon, Lyon, France; ^3^Carl Sagan Center for Research, The SETI Institute, Mountain View, CA, United States

**Keywords:** lipid biomarkers, El Tatio geyser field, hot springs, early life analog, silica deposits on Mars, DNA sequencing analysis, paleobiology, astrobiology

## Abstract

Hydrothermal systems and their deposits are primary targets in the search for fossil evidence of life beyond Earth. However, to learn how to decode fossil biomarker records in ancient hydrothermal deposits, we must first be able to interpret unambiguously modern biosignatures, their distribution patterns, and their association with physicochemical factors. Here, we investigated the molecular and isotopic profile of microbial biomarkers along a thermal gradient (from 29 to 72°C) in a hot spring (labeled Cacao) from El Tatio, a geyser field in the Chilean Andes with abundant opaline silica deposits resembling the nodular and digitate structures discovered on Mars. As a molecular forensic approach, we focused on the analysis of lipid compounds bearing recognized resistance to degradation and the potential to reconstruct the paleobiology of an environment on a broader temporal scale than other, more labile, biomolecules. By exploiting the lipid biomarkers’ potential to diagnose biological sources and carbon fixation pathways, we reconstructed the microbial community structure and its ecology along the Cacao hydrothermal transect. The taxonomic adscription of the lipid biomarkers was qualitatively corroborated with DNA sequencing analysis. The forensic capacity of the lipid biomarkers to identify biosources in fresh biofilms was validated down to the genus level for *Roseiflexus*, *Chloroflexus*, and *Fischerella*. We identified lipid biomarkers and DNA of several new cyanobacterial species in El Tatio and reported the first detection of *Fischerella* biomarkers at a temperature as high as 72°C. This, together with ecological peculiarities and the proportion of clades being characterized as unclassified, illustrates the ecological singularity of El Tatio and strengthens its astrobiological relevance. The Cacao hydrothermal ecosystem was defined by a succession of microbial communities and metabolic traits associated with a high- (72°C) to low-(29°C) temperature gradient that resembled the inferred metabolic sequence events from the 16S rRNA gene universal phylogenetic tree from thermophilic to anoxygenic photosynthetic species and oxygenic phototrophs. The locally calibrated DNA-validated lipidic profile in the Cacao biofilms provided a modern (molecular and isotopic) end member to facilitate the recognition of past biosources and metabolisms from altered biomarkers records in ancient silica deposits at El Tatio analogous to Martian opaline silica structures.

## Introduction

Hydrothermal springs are terrestrial environments characterized by extreme environmental conditions, such as high water temperature, sometimes several tens of degrees above the mean air temperature, pH values ranging from acidic to alkaline, or hydrothermal fluids often concentrated in noxious elements, such as Hg, Sb, B or As (Rothschild and Mancinelli, 2001). However, despite their apparent inhospitality, terrestrial hydrothermal springs are recognized habitats for microbial life on Earth (Brock, 1978) and are of scientific interest for the study of extremophilic microbial communities that resist and adapt to the extreme environments. In addition, terrestrial hydrothermal springs are credible candidate sites for the origin of life (Deamer and Georgiou, 2015; Damer and Deamer, 2020; Van Kranendonk et al., 2021), where the abundant supply of geothermal energy and nutrients (including B, Zn, Mn, and K, in addition to C, H, N, O, P, and S), as well as the alternate of wet-dry cycles, may have favored the prebiotic synthesis of complex organic compounds (Deamer and Georgiou, 2015). Thus, the colonization of geothermal environments by microbial communities attracts the interest of biogeochemists and astrobiologists to understanding the early evolution of the biosphere on Earth and the implications for a hypothetical development of life elsewhere in the Solar System.

Furthermore, terrestrial hydrothermal systems are also strategic targets to search for signs of past life since they accumulate deposits with potential for biosignatures preservation. Due to the rapid cooling and evaporation of silica-saturated fluids, siliceous sinters precipitate rapidly as amorphous silica (i.e., opal-A), allowing the preservation of microfossils, textural biosignatures, and organic biomolecules from the microbial communities that could have inhabited the silicifying hot spring waters (e.g., Campbell et al., 2015; Teece et al., 2020; Wilmeth et al., 2021). In addition, the silica surface provides a physical shield that protects against the high-intensity UV radiation, thus, favoring the preservation of biological remnants entombed within by avoiding extensive cellular damage (Phoenix et al., 2006). Siliceous sinter occurs throughout the rock record up to 3.5 Ga and includes the earliest evidence for life in non-marine environments (Djokic et al., 2017).

Opaline sinter deposits of ∼3.8 Ga that argue for the existence of hydrothermal activity in Hesperian times are also preserved on Mars (e.g., Gusev crater or Syrtis Major), constituting targets for future investigations of sedimentology and potential astrobiological signatures (Skok et al., 2010; Ruff and Farmer, 2016; Ruff et al., 2019). Thus, since proposed by Walter and Des Marais (1993), siliceous hydrothermal spring deposits are considered key Martian astrobiological targets because of their high habitability potential and ability to capture and preserve biosignatures (Walter and Des Marais, 1993; Cady et al., 2018; Teece et al., 2020). However, while physical access to opaline silica samples from Mars is unattainable at this stage, an indirect exploration through the analysis of analogous sinter deposits on Earth appears as a good alternative (e.g., Campbell et al., 2015).

From the extensive catalog of hydrothermal systems on Earth (e.g., Krýsuvik, Hveragerdi, Hveravellir or Namafjäll in Iceland; Octopus Spring, Mushroom Spring, Chocolate Pots or Fountain Paint Pots in Yellowstone National Park in the United States; Uzon Caldera, Geysir Valley, or Pauzhetka hot springs in Kamchatka, Russia; Umukuri, Tahunaatara, Mangatete, Te Kopia or Orakei Korako in the Taupo Volcanic Zone in New Zealand; Black Water, Gaet’ale, or other unnamed springs in the Dallol region in Ethiopia, etc.), the El Tatio geyser field in the Chilean Andes represents one of the best Martian analogs (e.g., Ruff and Farmer, 2016) because of the number of features resembling the Early Mars. Such common features include the high elevation of El Tatio (4,320 masl), which makes the Andean geyser field a unique environment with harsh conditions similar to some on Mars (Cabrol et al., 2007, 2018), such as an intense surface ratio of UV radiation (Cabrol et al., 2014), a large daily thermal oscillation, or high atmospheric dryness (i.e., a precipitation rate of 100 mm per year) (Fernández-Turiel et al., 2005). Thus, the El Tatio geyser field is a unique scenario to investigate the distribution of microbial life in high-altitude extreme conditions and to learn about the preservation of biosignatures in sinter deposits that resemble opaline silica outcrops described on Mars.

However, to interpret properly the information enclosed in old sinter deposits from El Tatio, we need to first understand the modern biomarker record and its preservation over time, and, therefore, it is crucial to know what biological sources contribute to it locally. Learning to interpret in detail modern biomarker records is relevant for recognizing past biosources and metabolic features in geological samples, where the preferential degradation of more labile biomolecules (e.g., DNA or proteins; Sánchez-García et al., 2020, 2021; Lezcano et al., 2022) makes the lipid remnants one of the most suitable biomarkers as their hydrocarbon skeletons can retain information for billions of years (Brocks et al., 2005). While the microbial diversity in the hydrothermal region of El Tatio is largely known by studies based on petrographic examinations of siliceous sinter deposits (Fernández-Turiel et al., 2005), *in situ* thermal imaging of microbial mats (Dunckel et al., 2009), electron microscopy of terrace sinter, and cyanobacterial biomass (Phoenix et al., 2006), or by microscopical analysis and DNA sequencing of partially silicified microbial communities (Barbieri et al., 2014; Wilmeth et al., 2021), little has been done by exploring highly resistant lipid biomarkers (Sánchez-García et al., 2019; Teece et al., 2020).

In active hydrothermal systems, the water temperature plays a key role in determining the composition and distribution of microbial mats or biofilms (Purcell et al., 2007) that may induce the biological precipitation of silica sinter (microbial silicification) from metabolic biochemistry (e.g., oxygenic photosynthesis or respiration) (Konhauser et al., 2004; Dupraz et al., 2009), although the exact role of microbes in the formation of siliceous sinters remains unclear (Gong et al., 2021; Jones, 2021). To interpret biological fingerprints in silicified/fossilized deposits of sinter, we need to understand well the effect of the temperature on the distribution of modern microbial communities in siliceous-rich hydrothermal springs. In this study, we investigated the molecular and isotopic composition of eight biofilms growing at different temperatures in a hot spring from El Tatio, dubbed the “Cacao” stream. By characterizing the lipid biomarkers profile from 29 to 72°C, we aimed to capture the transition of biological sources and prevailing metabolisms, with the increasing temperature to reconstruct the associated biofacies along the hydrothermal transect. We chose lipid biomarkers as a forensic tool because of the generally much higher resistance to degradation of lipids compared to other biomolecules (Sánchez-García et al., 2020, 2021; Lezcano et al., 2022), which makes them applicable to decoding fossil counterparts of silica sinter deposits within a wide time framework (Walter and Des Marais, 1993; Cady et al., 2018; Teece et al., 2020). We assessed the precision of the taxonomic assignation based on the organismic-ubiquitous lipid biomarkers by analysis of small subunit ribosomal ribonucleic acid (SSU rRNA) sequencing. The qualitatively validated forensic capacity of the lipid biomarkers to detect microbial biosources in the Cacao biofilms provided a fresh molecular and isotopic end member for interpreting fossil biomarkers in ancient silica records in El Tatio and analogous siliceous deposits on Mars.

## Materials and Methods

### Description of the Study Area

**FIGURE 1:**
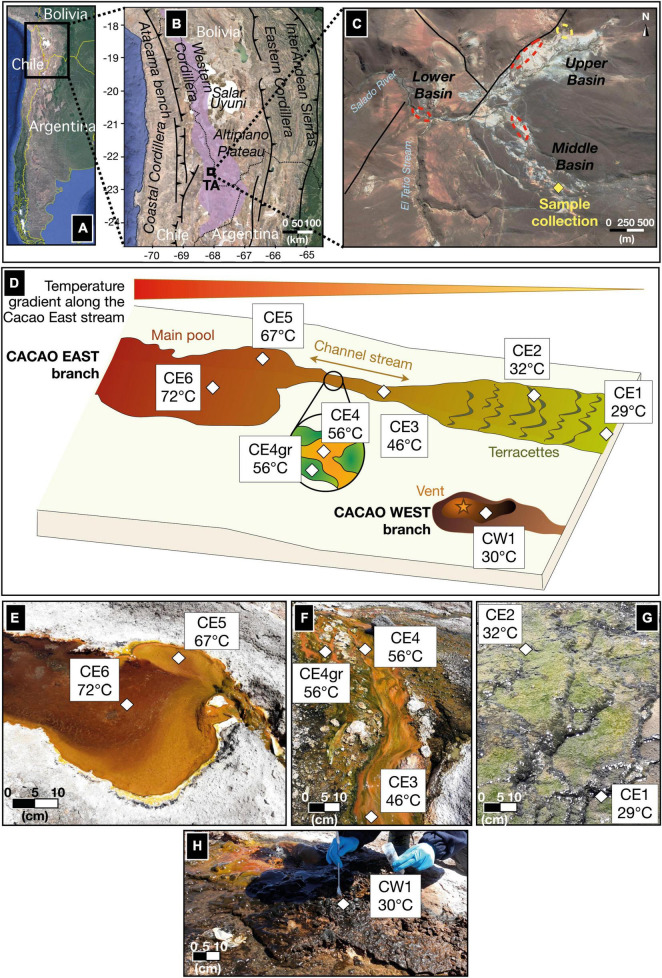
A study site and a sampling area along the Cacao hydrothermal stream in the geothermal region of El Tatio. **(A)** Location of the El Tatio geyser field in northern Chile; **(B)** the black rectangle zooms in the Chilean Altiplano, where the Andean modern volcanic arc (a.k.a. Altiplano-Puna Volcanic Complex or APVC) is located (the purple area). The regional fault system, corresponding to sectors of contractional tectonics, makes the APVC a very highly uplifted area, explaining the high elevation of the El Tatio (TA) area (Lucchi et al., 2009). **(C)** The black square marked TA represents the inset view of El Tatio as a *Google Earth* image, showing the three basins, comprising the hydrothermal system (modified from Muñoz-Saez et al., 2018) and the location of the Cacao stream (yellow diamond), where the eight biofilm samples were collected. Local faults (black lines) allow hydrothermal circulations and explain part of the repartition of the primary areas with geysers and spouters (red dashed circles) or mud pools and volcanoes (yellow dashed circles). **(D)** A sketch of the hydrothermal transect (from 72°C to 29°C) studied along the Cacao stream, with seven biofilm samples collected from the East branch (CE) and one from the West branch (CW). **(E–(H)** a close view of the seven CE biofilms along the Cacao stream; **(E)** the vent pool, **(F)** the mid-apron channel, and **(G)** distal-apron *terracetes*. **(H)** A close view of the eighth biofilm (CW1) collected from the West branch of the Cacao stream.

El Tatio geyser field (22°20′S 68°01′W), located in the modern volcanic arc of Central Andes in the Altiplano plateau in northern Chile (Figures 1A,B), is the largest known geyser field in the southern hemisphere. The hydrothermal area comprises more than 80 active geysers and thermal manifestations that expand over 30 km^2^ at elevations from 4,200 to 4,600 m.a.s.l. (Glennon and Pfaff, 2003; Fernández-Turiel et al., 2005). Hydrothermal fluids are mostly meteoric, with minor magmatic components. The fluids rich in silica generate local silica deposits on the surface that overlaps the geological sequence composed of ignimbrite, dacitic/andesitic lavas, and glacial and alluvial deposits (Lucchi et al., 2009). The hydrothermal area is divided into three distinct basins: upper, middle, and lower (Figure 1C); in all of them thermal features like geysers, fumaroles, geothermal springs, and mud volcanoes can be encountered (Muñoz-Saez et al., 2018).

The hydrothermal springs of El Tatio hold diverse microbial communities with the presence of photosynthetic thermophilic bacteria (e.g., *Chloroflexus* like), *Cyanobacteria* (e.g., *Oscillatoria*, *Nostoc*, *Phormidium, Leptolyngbya*, and *Calothrix*) and diatoms deduced from microscopic observations and thermal imaging (Fernández-Turiel et al., 2005; Phoenix et al., 2006). More recently, the presence of eukaryotic golden and green algae, as well as *Firmicutes* (*Bacillales* and *Clostridiales*), *Proteobacteria* (*Rhodobacteriales*, *Rhizobiales*, *Burkholderiales*, and *Desulfuromonadales*), *Bacteroidetes*, *Planctomycetes*, *Verrumicrobia*, or *Actinobacteria* (*Actinomycetales*) has also been reported from the use of DNA sequencing analysis on sinter deposits (Barbieri et al., 2014; Sánchez-García et al., 2019; Gong et al., 2020; Wilmeth et al., 2021).

### Sample Collection

Fresh biofilms from a hydrothermal stream in the middle basin of the El Tatio geyser field were sampled in November 2018 during a field campaign funded by the NASA Astrobiology Institute (NAI) within the NAI CAN7 project (ref. 13-13NAI7_2-0018). In total, eight biofilm samples were collected along the proximal to the distal apron of the hydrothermal stream named by our team as Cacao, along a transect of 10–12 m in length, covering a temperature gradient from 72°C in the hot spring pool to 29°C further down in the cooler *terracettes* (Figure 1D). The water pH was measured *in situ* and observed to vary little along the relatively small transect (i.e., from 6.9 to 7.2; Table 1), so the temperature was considered the main variable environmental factor. Two samples were collected from the upstream hot spring pool, CE6 (72°C) and CE5 (67°C) (Figure 1E), and three from the channel flowing down the pool, where water temperature decreases from 56°C (CE4 and CE4gr) to 46°C (CE3) (Figure 1F). Both CE4 and CE4gr were collected from the same site and at the same temperature, but their different aspects suggested a somehow different microbial composition (Figure 1D), that is, filaments dominantly orange (CE4) or green (CE4gr). Finally, two more biofilms, CE2 (32°C) and CE1 (29°C) were collected from the downstream *terracettes* formed by the precipitation of silica sinter as the water cools down at lower locations in the distal apron (Figure 1G). An additional biofilm was sampled from a parallel branch of the hydrothermal stream, the so-called Cacao West, at a temperature of 30°C (i.e., CW1). We added this sample to the set because of its different aspects (i.e., black; Figure 1H) compared to its temperature counterpart in Cacao East (i.e., CE1). See also Table 1.

**TABLE 1:** Location and description of the fresh biofilms studied along the Cacao hydrothermal stream, in the El Tatio middle basin.

Sample name^a^	Site latitude (S)	Site longitude (W)	Altitude (m)	Location on the stream	Sample visual description	Temperature (°C)	pH
CE6	22°21.022′	68°0.483′	4328	Upstream Cacao East, mean hot spring pool	Rusty colored biofilm with abundant mineral grains	72	6.96
CE5	22°21.022′	68°0.483′	4328	Upstream Cacao East, edge of the hot spring pool	Green borders over orange base with stream tufts	67	7.07
CE4	22°21.022′	68°0.483′	4328	Cacao East channel stream, upper half	Green-orange biomass	56	6.96
CE4gr	22°21.022′	68°0.483′	4328	Cacao East channel stream, upper half	Intense green biomass	56	6.96
CE3	22°21.020′	68°0.485	4327	Cacao East channel stream, lower half	Red-orange biomass	46	6.95
CE2	22°21.019′	68°0.486′	4327	Downstream Cacao East, on *microterracetes* surface	Bright green biomass	32	7.18
CE1	22°21.017′	68°0.485′	4326	Downstream Cacao East, furthest spot from the hot spring pool	Greenish-brown biomass	29	7.13
CW1	22°21.025′	68°0.480′	4328	Upstream Cacao West, outer layer around the vent	Blackish-brown biomass	30	7.15

*^a^The sample names are built according to a code (CE or CW) that refers to the stream branch (East or West, respectively) from which the samples were collected.*

At each temperature, a total sample of 15-100 g was collected from different spots (*n* = 3–5) on the microbial mats, combined to assure a representative sampling. The biofilm samples were retrieved with ethanol-clean spatulas and spoons, wearing a clean white suit, booties, and nitrile gloves to avoid contamination. They were stored in solvent-clean polypropylene containers and kept cold (i.e., 4°C) until arrival at the *Centro de Astrobiología* of Madrid, where they were frozen at –20°C. Before analysis, the samples were freeze-dried, and between 0.3 and 20 g of dry mass were available for the different biogeochemical analyses.

### Bulk Geochemistry Analysis

The stable carbon isotopic composition (δ^13^ C) of the biomass was measured on the bulk biofilm samples with isotope-ratio mass spectrometry (IRMS), following USGS methods (Révész et al., 2012). Briefly, subsamples of the freeze-dried biofilms (300 mg) were homogenized by grinding with a corundum mortar and pestle (except for sample CE4gr that had not enough material for all analyses). Subsequently, HCl was added to remove carbonates, and we let it equilibrate for 24 h, adjusting, then, the pH to neutral values with ultrapure water. Afterward, the residue was dried in an oven (50°C) for 72 h or until constant weight, and then it was analyzed by IRMS (MAT 253, Thermo Fisher Scientific, Waltham, MA, United States). The δ^13^C values were reported in the standard per mil notation using three certified standards (USGS41, IAEA-600, and USGS40), with an analytical precision of 0.1‰. The content of total organic carbon (TOC%) was measured with an elemental analyzer (HT Flash, Thermo Fisher Scientific, Waltham, MA, United States) during the stable isotope measurements.

### Lipid Biomarkers Extraction, Fractionation, and Analysis

Depending on the available amount of sample, between 0.5 and 2 g of freeze-dried sample were extracted with a mixture of dichloromethane and methanol (3:1, v/v) to obtain a total lipid extract (TLE) by an ultrasonic bath (further details in Sánchez-García et al., 2018). A mixture of three internal standards (tetracosane- D_50_, myristic acid-D_27_, and 2-hexadecanol) was added to the samples before the extraction to allow for the quantification of compounds in each lipidic fraction, namely, apolar, polar, and acidic. The TLE has concentrated to ca. 2 ml by rotary evaporation, and elemental sulfur was removed overnight with activated copper. Then, the clean extract was hydrolyzed overnight with methanolic KOH (6% MeOH) at room temperature. *n*-Hexane was added to the hydrolyzed TLE to obtain the neutral fraction through liquid-liquid extraction. The remaining lipidic extract was then acidified with HCl (37%) to remove K^+^ from the solution by precipitation of KCl, allowing the recovery of the liberated carboxylic groups by liquid-liquid extraction with *n-*hexane (acidic fraction). Further separation of the neutral fraction into apolar (hydrocarbons) and polar (alcohols) was conducted by using activated alumina (Sánchez-García et al., 2019). Before analysis by gas chromatography linked to mass spectrometry (GC-MS), both acidic and polar fractions were derivatized with BF_3_ in methanol (the fatty acids) and with N, *O*-bis (trimethylsilyl) trifluoroacetamide (the alcohols) to increase the volatility of their carboxyl and hydroxyl groups by transformation into fatty acid methyl esters (FAME) and trimethylsilyl derivates, respectively.

The three lipid polarity fractions (apolar, acidic, and polar) were analyzed by GC-MS using a 6850 GC system, coupled to a 5975 VL MSD, with a triple-axis detector (Agilent Technologies, Santa Clara, CA, United States), with electron ionization at 70 eV and scanning from m/z 50 to 650. For the apolar fraction, the oven temperature was programmed from 40°C to 150°C at 15°C⋅min^–1^ (held 2 min) to 255°C at 5°C⋅min^–1^ (held 20 min), and then to 300°C at 5°C⋅min^–1^ (held 10 min). For the polar and acidic fractions, the oven temperature was programmed from 50°C to 130°C at 20°C⋅min^–1^ (held 2 min) and to 300°C at 6°C⋅min^–1^ (held 20 min or 25 min for the acidic or polar fraction, respectively). The injector temperature was 290°C, the transfer line was 300°C and the MS source at 240°C. Compound identification was based on the comparison of mass spectra and/or reference materials using the NIST library provided by the MSD ChemStation software. For quantification, we used external calibration curves of *n-*alkanes (C_10_ to C_40_), *n-*fatty acids as FAME (C_8_ to C_24_), and *n-*alkanols (C_10_, C_14_, C_18_, and C_22_). All chemicals and standards were supplied by Sigma Aldrich. The recovery of the internal standards averaged 70 ± 21%.

### Compound-Specific Isotope Analysis

Compound-specific isotope analysis (CSIA) of the lipid biomarkers was performed on the three extracted polarity fractions to determine individual δ^13^C values assignable to carbon fixation pathways (Supplementary Text 1). The carbon isotopic composition of individual lipids was measured by coupling gas chromatography-mass spectrometry (Trace GC 1310 ultra and ISQ QD MS) to an isotope-ratio mass spectrometry system (MAT 253 IRMS, Thermo Fisher Scientific, Waltham, MA, United States). The conditions for the GC analysis were identical to those used for the molecular analysis of the polar fraction, whereas those for the IRMS analysis included an electron ionization of 100 eV, Faraday cup collectors of m/z 44, 45, and 46, and a temperature of the CuO/NiO combustion interface of 1,000°C. The samples were injected in a splitless mode, with an inlet temperature of 250°C, and helium as carrier gas at a constant flow of 1.1 ml⋅min^–1^. The δ^13^C values of the individual lipids separated by GC were calculated using CO2 spikes of known isotopic composition, introduced directly into the MS source, three times at the beginning and the end of every run. Reference mixtures from Indiana University (US) of the known isotopic composition of *n*-alkanes (A6) and FAMEs (F8) were run every four samples to check the accuracy of the isotopic ratio determined by GCMS-IRMS. The δ^13^C data for individual carboxylic acids were calculated from the resulting FAME values by correcting them for the one carbon atom added in the methanolysis (Abrajano et al., 1994). Similarly, the values from the trimethylsilyl derivates in the polar fraction were also corrected for the 3 carbons added in the derivatization process.

### DNA Extraction, PCR Amplification, and DNA Sequencing

In addition to the molecular and isotopic characterization of the lipid biomarkers, the eight biofilms were subjected to genetic analysis to determine with precision the taxonomic composition of the hydrothermal samples. This complimentary analysis aimed to test the reliability and specificity of the lipid biomarkers to infer biological sources and to attempt taxonomical affiliations.

#### Biofilms DNA Extraction

Genomic DNA of the freeze-dried biofilms was extracted using a DNeasy Power Biofilm Kit (QIAGEN, Hilden, Germany), following the manufacturer’s instructions with several modifications. Samples from 0.1 to 0.25 g (depending on the available amount of material for each sample) were split into two subsamples to increase the DNA extraction yield. Based on previous tests, we considered that this amount of sample was sufficient for efficient DNA extraction and that it was representative of the whole biofilm as the subsamples (i.e., extraction replicates) were taken from the homogenized freeze-dried samples. Then, from ca. 550 μL to 1 ml of MBL solution was added to each biofilm material to prevent the total absorption of the liquid by the freeze-dried biofilms. Moreover, the samples were incubated two times with 100 μL of IRS solution on ice to efficiently remove the high amount of non-DNA organic and inorganic material in the biofilms. The final subsample DNA extractions were combined in the MB Spin Columns, and several additional centrifugation steps at 20,000 × *g* were carried out to remove the ethanol completely before recovering the DNA with 50 μL of sterile water. Negative control of the kit without a sample was also performed. DNA concentrations were determined using the Qubit dsDNA BR Assay kit (Invitrogen, Thermo Fisher Scientific, Waltham, MA, United States), and DNA extracts were stored at –20°C until sequencing analysis at the genomic service of *Fundación Parque Científico de Madrid* (FPCM, Spain).

#### PCR Amplification of Small Subunit Ribosomal Ribonucleic Acid Genes and Sequencing

The distribution of bacteria, archaea, and eukarya, as well as the specific cyanobacterial community, was analyzed by the construction of amplicon libraries and sequenced on an Illumina MiSeq sequencer (Illumina Inc., San Diego, CA, United States) at the genomic service at FPCM. The bacterial 16S rRNA V3–V4 hypervariable gene region was amplified with the primer pair Bakt_341-F/Bakt_805- R (Herlemann et al., 2011). The archaeal 16S rRNA V2–V3 hypervariable gene region was amplified with the primer pair Arch1F/Arch1R (Cruaud et al., 2014), and the eukaryotic 18S rRNA V4–V5 hypervariable gene region was amplified with the primer pair 563F/1132R (Hugerth et al., 2014). These primers have been previously used to characterize the microbial communities in sinter samples from El Tatio (Sánchez-García et al., 2019) and other geothermal environments (Lezcano et al., 2019). The 16S rRNA gene of cyanobacteria was amplified with the specific primer pairs CYA359F/CYA781Ra and CYA781Rb (Nübel et al., 1997) in separate reactions. These specific cyanobacterial primers are widely used to identify cyanobacteria in different environments (e.g., Dorador et al., 2008; Azúa-Bustos et al., 2011; Mehda et al., 2021). Further information about the PCR conditions is available in the Supplementary Text 2. The negative control showed absence of gene amplification and then was removed for the sequencing analysis. The PCR of the eukaryotic community showed gene amplification in CE1, CE2, and CW1 and was negative for the rest of the samples. Thus, only 18S rRNA gene amplicons from CE1, CE2, and CW1 were sequenced. Final amplicon pools were denatured before seeding on a flow cell and sequenced using the MiSeq Reagent kit v3 (Illumina, Inc., San Diego, CA, United States) in a 2 x 300 pair-end sequencing run on an Illumina MiSeq sequencer.

Raw sequences of bacteria (1,918,397), archaea (915,696), eukarya (479,652), and cyanobacteria (478,079 with primer pairs CYA359F/CYA781Ra, and 456,825 with primer pairs CYA359F/CYA781Rb) were processed in Mothur software v.1.45.3 (Schloss et al., 2009), following the MiSeq SOP pipeline (Kozich et al., 2013). Forward and reverse reads were merged and quality-filtered by removing the (i) reads below 400 bp for bacteria, 300 bp for archaea, 500 bp for eukarya, and 400 bp for cyanobacteria, (ii) ambiguous nucleotides, (iii) homopolymers longer than 8 bp and (iv) chimeras identified with VSEARCH (Rognes et al., 2016). Final sequences were clustered into OTUs at 97% similarity, and taxonomic assignments were performed against the SILVA database (v. 132; Quast et al., 2013). Singletons and sequences that were assigned to non-bacterial, non-archaeal, non-eukaryotic, and non-cyanobacterial entities in their respective gene libraries were removed to avoid misinterpretations. This final data screening resulted in the elimination of 0.62% of the sequences in the bacterial library (0.6% were singletons), 96.4% in the archaeal library (96.1% were bacterial sequences), 9% in the eukaryotic library (all were singletons), 35% in the cyanobacterial library with the primer pairs CYA359F/CYA781Ra (34.5% were other bacteria different from *Cyanobacteria*) and 29.8% in the cyanobacterial library, with the primer pairs CYA359F/CYA781Rb (29.3% were other bacteria different from *Cyanobacteria*). Then, the resulting cyanobacterial reads were combined to get a single data set.

Shannon-Wiener, Simpson, and Fisher diversity indices were calculated with the vegan package (Oksanen et al., 2020) in R 4.1.3 as α-diversity estimators for the community composition of bacteria, cyanobacteria, archaea, and eukaryotes in the El Tatio biofilms.

## Results

### Bulk Geochemical Characterization of the Eight Biofilms Collected From Cacao Along a Thermal Gradient

All biofilm samples except CE4gr, which did not have sufficient material for bulk characterization, showed a biomass content (measured as TOC) from 0.46 to 27% of the dry weight (DW), and values of the bulk δ^13^C ratio, ranging from –19.5‰ to –4.7‰ (Table 2 and Supplementary Figure 1). The samples from 46 to 56°C were the most biomass-rich biofilms (i.e., CE3 and CE4), and that from 72°C (CE6) were the poorest (Supplementary Figure 1). In the latter sample, the biomass was particularly depleted in ^13^C (i.e., showed the most negative δ^13^C values), whereas, in CW1, CE1, CE2, and CE5, the biomass was rather enriched (i.e., showed less negative δ^13^C values).

**TABLE 2:** Bulk geochemical characterization of the biofilms from the West and East branches of the Cacao hydrothermal stream (for CE4gr, there was no sufficient material for conducting the bulk geochemistry analysis).

Samples	CW1		CE1		CE2		CE3		CE4		CE5		CE6	
Temperature	30°C		29°C		32°C		46°C		56°C		69°C		72°C	
	Mean	std	Mean	std	Mean	std	Mean	std	Mean	std	Mean	std	Mean	std
δ^13^C (‰)	–8.5	0.08	–5.4	0.16	–6.9	0.07	–10.5	0.09	–11.1	0.10	–4.6	0.03	–19.4	0.07
TOC (%)	3.7	0.07	1.5	0.13	5.4	0.20	17	0.20	27	0.24	4.2	0.02	0.5	0.01

*The stable-carbon isotopic ratio (δ^13^C) of the total organic carbon (TOC) was measured on replicates (n = 4).*

### Molecular Distribution of Lipid Biomarkers Along the Cacao Hydrothermal Transect

**FIGURE 2:**
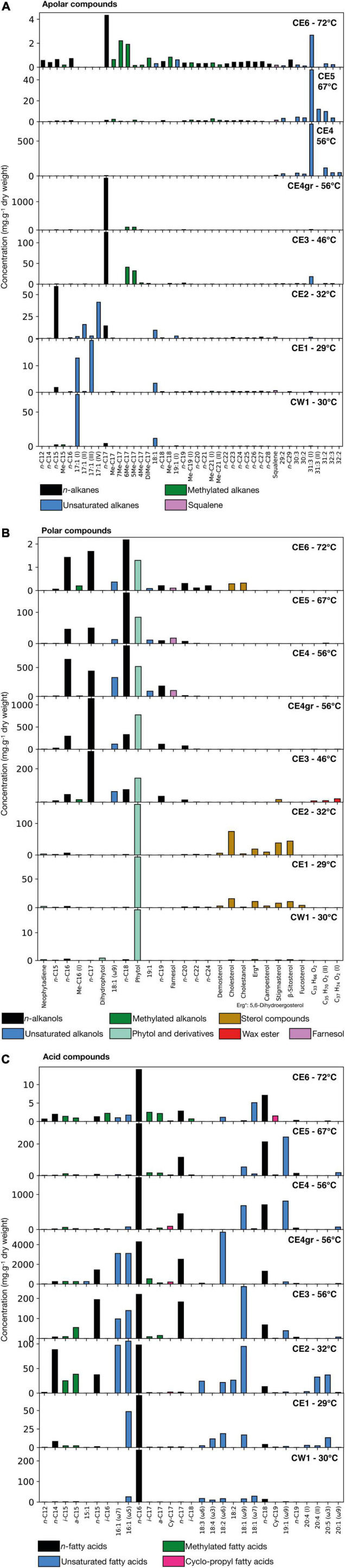
Concentration (mg⋅g^–1^ of dry weight) of the different lipid compounds identified in the eight Cacao biofilms along three polarity fractions; **(A)** apolar, **(B)** polar, and **(C)** acidic. Within each fraction, only the main compounds that represent more than 1% of the total mass concentration are plotted. Note that the vertical scale is different for each biofilm as the total concentration of organic matter varied between samples, and we focus on the relative distribution of lipid compounds. Different colors are used to represent different types of molecules (linear and saturated or *normal* chains, chains with double bonds or branches, aromatics, etc.). In the three panels, “unsaturated” compounds stand for chains with double bonds represented in the N:M format, where N indicates the number of carbons composing the chain and M the number of double bonds (e.g., 18:1 is a chain of 18 carbons with one double bond). In **(B)** the “phytol and derivatives” category includes neophytadiene and dihydrophytol besides phytol. In **(C)** *iso* and *anteiso* compounds (*i*-/*a*-) are monomethylated chains with one methyl group at the N-1 or N-2 carbon, respectively. In **(C)**, the position of the double bond is defined using the omega notation, that is, the position starting from the final carbon. In **(C)** Cy-C_*N*_ corresponds to mid-chain cyclo-propyl compounds, with chains of N carbons. The raw mass chromatograms used for compound identification and building the bar plots are available in the Supplementary Figure 3.

The total concentration of lipidic compounds identified in the Cacao biofilms varied within the samples from 0.088 mg⋅g^–1^ in CE6 (72°C) to 28.3 mg⋅g^–1^ in CE4gr (56°C), relative to the dry weight (Supplementary Figure 2). Of the three polarity fractions isolated, the acidic fraction was the most abundant in all biofilms, particularly in the CE4gr sample, where its concentration represented fivefold that of the two other fractions (Supplementary Figure 2). A varied distribution of compounds was detected in each lipidic fraction of the biofilms (Supplementary Table 1 and Supplementary Figure 3), where those of concentration representing more than 1% of the total mass concentration are plotted in Figure 2. Beyond the absolute concentration of lipid compounds, what is really of interest in the lipid biomarker approach is the molecular distribution within lipid families and the relative abundance of compounds. Therefore, our analysis here will focus on the detection of molecular patterns associable with biological sources according to assignments described in the literature based on culture-based studies or molecular, isotopic, microscopic, or genetic analyses.

#### Apolar Lipid Fraction

The distribution of lipidic compounds from the apolar fraction varied among the eight biofilms at different temperatures (Figure 2A). In general, there was a relatively higher proportion of compounds of low molecular weight (LMW) (≤ 18 carbons) in the biofilms at temperatures of ≤56°C, while of high molecular weight (HMW) in the samples at higher temperatures. In particular, the three biofilms thriving at temperatures ≤ 32°C (i.e., CW1, CE1, CE2) showed a relative abundance of different isomers of heptadecene (i.e., 17:1), and pentadecane (*n*-C_15_), mostly in CE2 (Figure 2A). From 46 to 56°C (only CE4gr), the predominant compounds were heptadecane and to a minor extent, different methyheptadecanes, either mono- (Me-C_17_) or dimethyl- (DMe-C_17_) homologs. From CE4gr to CE4 at 56°C, the molecular distribution of apolar compounds shifted drastically from a relative abundance of LMW to HMW and polyunsaturated hydrocarbons (Figure 2A). A set of compounds from 29 to 33 carbons with mono-, di-, and tri-unsaturations (i.e., one, two, or three double bonds) with the dominance of hentriacontatriene (31:3) was similarly observed in CE4 and CE5. Finally, at 72°C, a mix distribution of all the above-mentioned compounds was found to compose the apolar fraction of CE6, with shared dominance of *n*-C_17_ and 31:3, a generalized presence of all saturated alkanes from 12 to 29 carbons, and a relatively abundant and varied distribution of Me-C_17_ and DMe-C_17_ homologs. This varied distribution of methylheptadecanes contrasted with those of the samples CE3 or CE4gr, which contained almost exclusively the 5Me-C_17_ and 6Me-C_17_ homologs. Squalene was also detected in the apolar fraction of all biofilms except CE4gr (Figure 2A), and the hopanoid diplotene was only found in CE1, CE2, CE3, and CE4gr (Supplementary Table 1).

#### Polar Lipid Fraction

In the polar fraction, two different distribution patterns were observed among the eight biofilms (Figure 2B). In samples from temperatures of ≤32°C, the molecular distribution exhibited clear dominance of phytol and, mostly, in CE1 and CE2, a relative abundance of diverse sterol compounds (mostly cholesterol, β-sitosterol, stigmasterol, campesterol, fucosterol, and 5,6-dihydro ergosterol). In contrast to phytol, the sterol compounds were no longer detected in biofilms above 46°C (except for CE6 at a very low concentration). In addition, at temperatures of ≥ 46°C, the dominant presence of phytol was accompanied by *n-*alkanols from 16 to 24 carbons and monounsaturated alkanols of 18 and 19 carbons (i.e., 18:1 and 19:1). Among the *n-*alkanols series, heptadecanoic (*n*-C_17_) showed the highest relative abundance in CE3 and CE4gr, whereas octadecanoic (*n*-C_18_) was the most abundant in CE4, CE5, and CE6 (Figure 2B). A series of wax esters with carbon chains from 33 to 38 carbons (Supplementary Table 1 and Supplementary Figure 4) was also detected in the polar fraction of mostly CE3 (Figure 2B), as well as in CE4gr, CE4, and CE5 (Supplementary Table 1). Finally, the sesquiterpene farnesol was detected only at temperatures ≥ 56°C (i.e., from CE4 to CE6).

#### Acidic Lipid Fraction

The acidic profiles of all biofilms showed a molecular distribution dominated by the presence of LMW compounds (≤ C_20_) (Figure 2C), in particular, hexadecanoic acid (*n*-C_16_). Other saturated and unsaturated compounds are distributed differently among the eight biofilms. For instance, the monounsaturated acids 16:1 (ω5 and/or ω7) and 18:1 (ω7 or mostly ω9) were relatively more abundant at temperatures of ≤ 56°C, whereas the heptadecanoic (*n*-C_17_) and octadecanoic (*n*-C_18_) acids were rather more abundant at temperatures of ≥ 46°C, or the monounsaturated 19:1 (ω9) from 56°C to 67°C (Figure 2C). The presence of polyunsaturated acids, such as 18:4 (ω3), 18:3 (ω6), or 20:4, and 20:5 (ω3) was limited to the biofilms at lower temperatures (i.e., ≤ 32°C), whereas mid-chain cyclo-propyl acids (mostly Cy-C_17_ and Cy-C_19_) were relatively more abundant at temperatures ≥ 56°C. In contrast, monomethylated acids with *iso* and *anteiso* configuration (i.e., chains with a methyl group in positions N-1 and N-2, respectively) distributed ubiquitously (mostly the *i/a*-C_15_ and *i/a*-C_17_ pairs), although with higher relative abundance in the biofilms at 32, 46, and 72°C (Figure 2C).

### Stable Carbon Isotopic Composition of Microbial Lipid Biomarkers Along the Cacao Hydrothermal Stream

**FIGURE 3:**
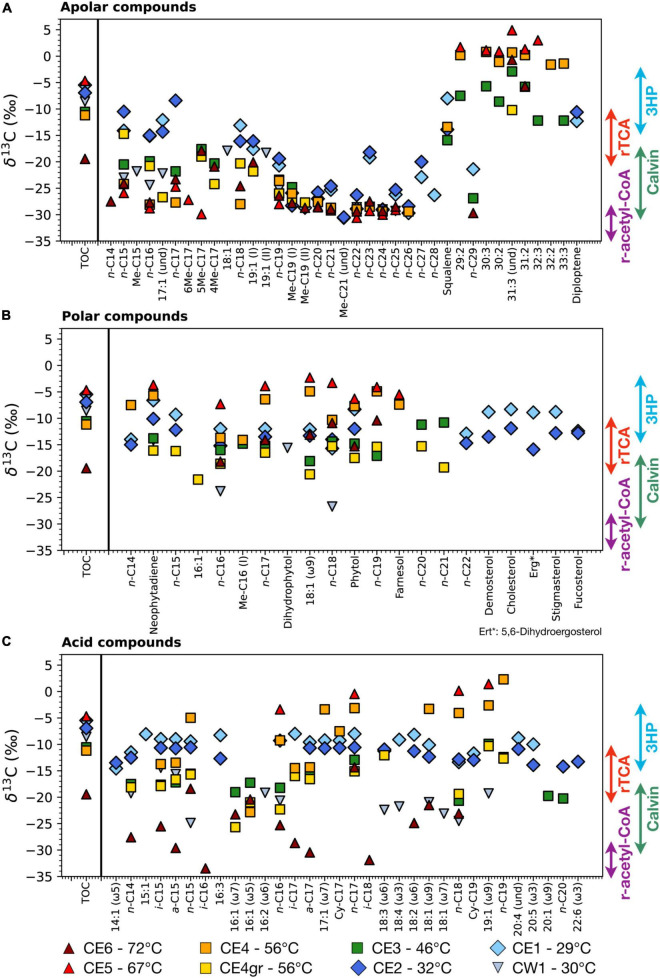
Compound-specific isotopic composition (δ^13^C) of the lipid compounds identified in the three polarity fractions; **(A)** apolar, **(B)** polar, and **(C)** acidic. The isotopic composition of the total biomass (δ^13^C_TOC_) is shown in each fraction for comparing the isotopic composition of the bulk cell material with that of individual lipid compounds. Colored arrows represent canonical biomass δ^13^C values associated with the four primary carbon fixation pathways (Preuß et al., 1989; van der Meer et al., 2000; Hayes, 2001). Note that individual lipid compounds are typically lighter (i.e., more negative δ^13^C) than the bulk organic matter, except for those synthesized through the reductive tricarboxylic acid (rTCA) cycle and, sometimes, the 3-hydroxypropionate (3HP) bicycle (Meyers, 1997; Jahnke et al., 2001).

The compound-specific carbon isotopic composition (δ^13^C) of all the identified lipid compounds ranged from –33.5‰ to +4.9‰ in the eight biofilms and showed different distribution patterns, depending on the temperature and the polarity fraction (Figure 3).

**FIGURE 4:**
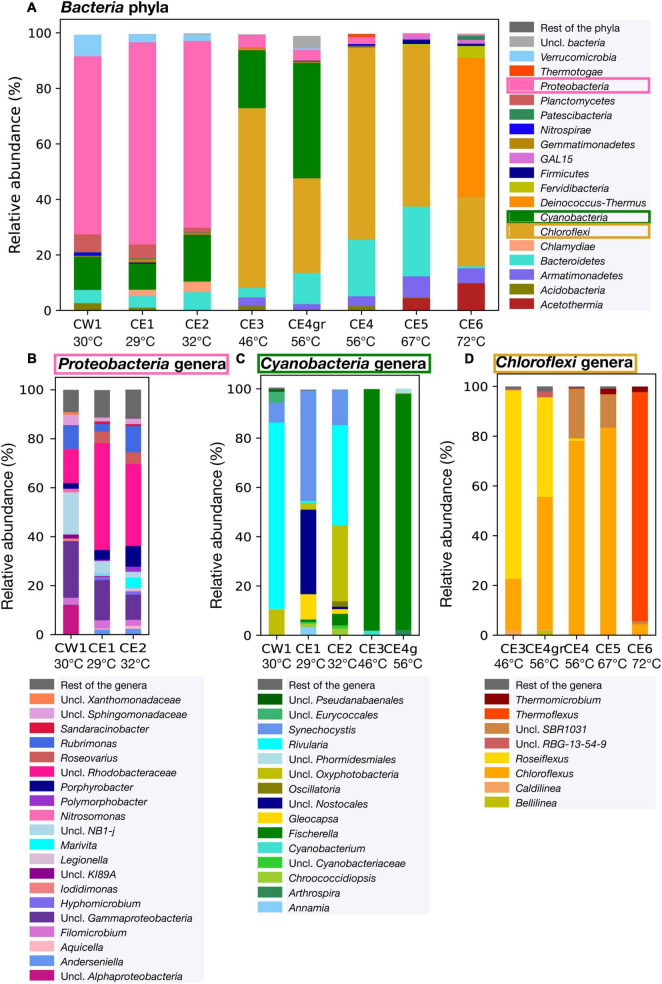
Relative abundance of the bacterial community composition in the eight Cacao biofilms based on DNA sequencing. **(A)** Relative abundance of the bacterial phyla within the eight biofilms. **(B)** Relative abundance of *Proteobacteria* genera in the low-temperature biofilms (≤ 32°C), where the phylum was the most abundant. **(C)** Relative abundance of *Cyanobacteria* in biofilms of low and mid-temperature (from 29 to 56°C) at the genus level based on the 16S rRNA gene amplified with the specific primers for *Cyanobacteria*. Only the cyanobacterial profiles of the low temperature biofilms are shown, as this phylum was relatively more abundant in those samples. **(D)** Relative abundance of *Chloroflexi* in the mid to high-temperature biofilms (from 46 to 72°C) at the genus level. *Uncl*, unclassified.

In the apolar fraction (Figure 3A), the values of the compound-specific δ^13^C varied from –30.6‰ to +4.9‰ and clustered into two general groups; (i) one of the relatively ^13^C-enriched values (i.e., δ^13^C from ca., –12‰ to + 4.9‰) largely corresponding to HMW poly-unsaturated alkanes (≥ 29 carbons), and (ii) another of relatively ^13^C-depleted values (i.e., from ca., –30.6‰ to –12‰) that included most of the remaining apolar compounds. In particular, *normal* alkanes of middle molecular weight (i.e., from 19 to 26 carbons) showed the most depleted δ^13^C values, mostly in CE6, CE5, and CE4 (Figure 3A), whereas those of LMW presented a more dispersed range of values, from as high as –8.4‰ in the *n*-C_17_ of CE2 to as low as –29.9‰ in the 5Me-C_17_ of CE5 (Figure 4A). Among the samples, the most ^13^C-enriched values were measured in the HMW compounds of CE4 and CE5 (i.e., 56 and 67°C).

The stable-carbon isotopic composition was relatively more homogeneous among the compounds in the polar fraction, where the δ^13^C values ranged only from –26.7‰ to –2.3‰ (Figure 3B). Similar to the apolar fraction, the samples CE4 (56°C) and CE5 (67°C) showed the most ^13^C-enriched δ^13^C values, whereas CE3 (46°C), CE4gr (56°C), and, mostly, CW1 (30°C) displayed the most ^13^C-depleted signatures.

In comparison, the range of variation of δ^13^C was broader in the acidic fraction (from –33.5‰ to +2.3‰), with values showing different distribution patterns among the eight biofilms (Figure 3C). The most enriched δ^13^C values (from –5‰ to + 2.3‰) were consistently found in compounds from CE4 and CE5, whereas the most depleted values were those of compounds (mostly *i*-/*a*-C_15_, *i*-/*a*-C_17_, *i*-C_16_, and *i*-C_18_) detected in the highest-temperature CE6 (from –14.3 to –33.5‰). The range of variance of the compound-specific δ^13^C was narrowest in the low-temperature CE1 and CE2 (from –14.6 to –8.‰), and relatively broader in CW1 (from –24.9 to –14.4‰), CE3 (from –20.7 to –9.9‰), and CE4gr (from –25.7 to –10.3‰).

### Taxonomic Analysis of the Cacao Biofilms Based on DNA Sequencing Data

Taxonomic analysis of the 16S rRNA gene sequences of the eight biofilms showed that bacterial and archaeal communities were present in all the biofilms except CE3, where no *Archaea* were detected after the quality-filtering of the sequences (all were *Bacteria*) (Supplementary Figure 5). Despite the identification of *Archaea* in the rest of the samples, they only represent 0.3% of the total sequences in the archaeal libraries (the rest were *Bacteria*), and, therefore, interpretations must be considered with caution. In contrast to *Bacteria* and *Archaea*, the 18S rRNA gene from *Eukaryota* was amplified only in the low-temperature biofilms (≤ 32°C), indicating detectable eukaryotic communities in CW1, CE1, and CE2.

In the bacterial community, the relative abundance of the phyla varied among the eight biofilms thriving at different temperatures in the hydrothermal stream (Figure 4A). In the low-temperature biofilms (≤ 32°C), *Proteobacteria* (recently renamed as *Pseudomonadota*) were largely predominant (> 60% of the bacterial sequences), with a lesser but still relevant representation of *Cyanobacteria* (from 9.4 to 16.7%). Among *Proteobacteria* (Figure 4B), unclassified (uncl.) *Rhodobacteraceae* (within the *Rhodobacterales* order) were the most represented in CE1 and CE2 (44 and 34% of the *Proteobacteria* sequences, respectively), followed by uncl. *Gammaproteobacteria* (10% and 16%), and uncl. *NB1-j* (2 and 5%). In CW1, the dominance of the uncl. *Gammaproteobacteria* (23% of the *Proteobacteria* sequences) was followed by a similar representation of uncl. *NB1-j* (17%), uncl. *Rhodobacteraceae* (14%), and uncl. *Alphaproteobacteria* (12%). Among *Cyanobacteria*, the genera *Rivularia*, uncl. *Oxyphotobacteria* and *Synechocystis* dominated in CW1 and CE2, while those of *Synechocystis*, uncl. *Nostocales*, and *Gloeocapsa* dominated in CE1.

In the biofilms from 46 to 56°C, the proportion of *Cyanobacteria* increased to 21% in CE3 and 42% in CE4gr (Figure 4A). In contrast to the low-temperature biofilms, the qualitative distribution of *Cyanobacteria* in CE3 and CE4gr was substantially more homogeneous (Figure 4C) and was dominated by *Fischerella* (> 95% of the cyanobacterial sequences).

Together with *Cyanobacteria*, the phylum of *Chloroflexi* (i.e., green non-sulfur bacteria) started to be the most representative of the bacterial communities in the biofilms from temperatures ≥ 46°C (Figure 4A), with a maximum (69%) at 56°C (i.e., CE4). Qualitatively, the distribution of *Chloroflexi* also changed at the genus level among the biofilms at different temperatures (Figure 4D). *Roseiflexus* and *Chloroflexus* were the most represented genera in CE3 (76 and 21%, respectively) and CE4gr (53 and 40%, respectively). The relative abundance of *Chloroflexus* increased in biofilms at higher temperatures, from 20% in CE3 (46°C) to a maximum of 83% in CE5 (67°C), whereas the relative abundance of *Roseiflexus* dropped drastically at 56°C from 40% in CE4gr to 1% in CE4 (Figure 4D). In CE4 (56°C) and CE5 (67°C), *SBR1031* was the second most represented genus after *Chloroflexus*. Finally, at the highest temperature (72°C), the genus *Thermoflexus* dominated the *Chloroflexi* community (92%), with the lower representation of *Chloroflexus* (4%) and *Thermomicrobium* (2%).

*Deinococcus-Thermus* was a phylum almost exclusively detected in CE6, where it represented 50% of the bacterial sequences (Figure 4A). Other bacterial phyla represented in smaller proportions in CE6 samples were *Acetothermia* (10%), *Armatimonadetes* (5%), *Fervidibacteria, Firmicutes* (now *Bacillota*), and *Planctomycetes* (all < 5%). While *Bacteroidetes* (now *Bacteroidota*) was ubiquitous in the Cacao biofilms majorly up to 67°C, where it showed its maximum relative abundance (25%), the distribution of *Verrumicrobia* and *Planctomycetes* was mostly restricted to the biofilms at temperature ≤ 32°C, and that of *Acetothermia* to those at temperature ≥ 67°C (Figure 4A).

Similar to the bacterial community, the distribution of *Archaea* and *Eukaryota* also exhibited differences among the biofilms at different temperatures (Supplementary Figure 5). In the archaeal community, *Crenarchaeota* was the only phylum identified in the biofilms CE5 (67°C), CE6 (72°C), and CW1 (30 °C), and the most abundant in CE4 (56°C) (Supplementary Figure 5A). In contrast, a mix of *Thaumarchaeota, Euryarchaeota*, and *Nanoarchaeota* composed the archaeal community of CE1 (29°C), and that of CE2 (32°C) and CE4gr (46°C) was exclusively comprised by *Nanoarchaeota*. Concerning the *Eukaryota* domain, the 18S rRNA genes could only be amplified in the low-temperature biofilms CW1, CE1, and CE2 (Supplementary Figure 5B), with most of the sequences in CE1 and CE2 related to uncl. *Eukaryota* and those in CW1 to the *Nematoda* (55%) and *Arthropoda* (37%) phyla.

Richness (number of different OTUs) and diversity indices (Shannon-Wienner, Simpson, and Fisher) showed variations among samples, reflecting a relationship with temperature (Table 3). Bacteria and eukaryotes showed the highest diversity and richness at the lowest temperatures (i.e., CW1, CE1, and CE2), whereas cyanobacteria also showed similar richness up to 56°C and a variable diversity with temperature. Archaea were hardly detected in the samples, except in CE6, where 19,064 sequences and 42 different OTUs were obtained, although mainly composed of a single OTU (85% order *Desulfurococcales*, phylum *Crenarchaeota*), which explains the low Simpson’s diversity value (0.26).

**TABLE 3:** Number of filtered sequences (Seqs), richness (number of different OTUs), and diversity indices (Shannon-Wiener or H′, Simpson, and Fisher) in the biofilm samples from the high-throughput sequencing of the 16S rRNA gene of bacteria, cyanobacteria, and archaea, and the 18S rRNA gene of eukaryotes.

		CW1	CE1	CE2	CE3	CE4gr	CE4	CE5	CE6
Bacteria	Seqs	144399	148819	125521	117441	169208	134093	111025	120713
	Richness	658	1268	1111	392	485	310	260	289
	*H*′	3.76	4.40	4.35	1.88	2.38	1.68	1.87	1.68
	Simpson	0.95	0.97	1.00	1.00	0.78	1.00	0.73	0.69
	Fisher	89.01	190.30	167.87	50.58	61.20	37.94	31.88	35.54
Cyanobacteria	Seqs	92627	87869	94367	90517	85339	19385	107	3576
	Richness	132	148	171	139	141	54	11	42
	*H*′	1.65	2.42	2.38	0.85	0.94	0.74	1.95	2.80
	Simpson	0.71	0.86	0.86	0.52	0.53	0.50	0.83	0.92
	Fisher	15.14	17.35	20.24	16.10	16.49	6.79	3.07	6.68
Archaea	Seqs	4	27	20	n.a.	160	91	245	19064
	Richness	3	4	2	n.a.	3	9	10	42
	*H*′	1.04	1.26	0.67	n.a.	0.72	1.64	1.03	0.66
	Simpson	0.63	0.69	0.48	n.a.	0.50	0.74	0.55	0.26
	Fisher	5.45	1.30	0.55	n.a.	0.52	2.48	2.10	5.11
Eukaryota	Seqs	41997	102884	47861	n.a.	n.a.	n.a.	n.a.	n.a.
	Richness	943	1787	1311	n.a.	n.a.	n.a.	n.a.	n.a.
	*H*′	2.16	1.86	2.63	n.a.	n.a.	n.a.	n.a.	n.a.
	Simpson	0.69	0.58	0.79	n.a.	n.a.	n.a.	n.a.	n.a.
	Fisher	171.26	307.21	249.07	n.a.	n.a.	n.a.	n.a.	n.a.

*“n.a.” indicates not available.*

## Discussion

### Biological Composition of the Fresh Biofilms Along the Cacao Hydrothermal Stream Based on Lipid Biomarkers

Lipids are cell membrane constituents with powerful diagnosis potential for several biological groups (i.e., biomarkers) that can be limitedly specific to some taxa (Summons et al., 2021). Their analysis provides identification of prevailing biological sources in an environmental sample and variable distribution patterns as a function of environmental factors. In the Cacao hydrothermal stream, we detected several lipid compounds that can be associated with diverse prokaryotic and eukaryotic biosources according to the literature (Table 4).

**TABLE 4:** Potential biological sources of lipidic molecules isolated from the Cacao stream biofilms.

Compound	Potential biological source^a^	References
**Hydrocarbons**		
17:1	*Cyanobacteria*	Shiea et al., 1990; Grimalt et al., 1992; Coates et al., 2014
*n-*C_17_	*Cyanobacteria*	Gelpi et al., 1970; Shiea et al., 1990; Coates et al., 2014
3/4/5/6/7Me-C_17_	*Cyanobacteria*	Shiea et al., 1990; Teece et al., 2020; Coates et al., 2014
Me-C_19_	*Cyanobacteria*	Kenig et al., 2005
Squalene	Most organisms, *Archaea* likely predominant	Grice et al., 1998
≥ 29:n	*Chloroflexi*	Shiea et al., 1991; van der Meer et al., 1999, 2001
Diploptene	*Cyanobacteria*, methanotrophic bacteria	Gelpi et al., 1970; Rohmer et al., 1984; Sakata et al., 1997
**Alcohols and Sterols**		
*n-*C_17_	*Cyanobacteria*	Bühring et al., 2009
Phytol	Phototrophs (plants, algae, *Cyanobacteria*, green sulfur and non-sulfur bacteria)	Didyk et al., 1978
Neophytadiene	Likely transformation product of phytol	Didyk et al., 1978
Dihydrophytol	Likely transformation product of phytol (from hydrogenation)	Didyk et al., 1978
Farnesol	Purple sulfur bacteria	Bühring et al., 2009
Cholesterol	Animals, microalgae, diatoms, red algae	Volkman, 2003
Demosterol	Precursor of cholesterol	Volkman, 2003
5,6-Dihydroergosterol	Possibly derived from ergosterol. Ascomycetes, basidiomycetes, unicellular green algae.	Nes and McKean, 1977; Weete et al., 2010
Stigmasterol	Higher plants (mostly) and microalgae	Volkman, 1986, 2003
Fucosterol	Microalgae, macroscopic brown algae, diatoms, detected in lacustrine *Bacillus* sp.	Patterson, 1972; Mercer et al., 1974
Stigmast-5-ene	Likely derived from stigmasterol (mostly higher plants and microalgae)	Volkman, 2003
Wax ester (C_33_ to C_37_)	*Chloroflexi* (*Chloroflexus aurantiacus* and relatives)	Shiea et al., 1991; van der Meer et al., 2000
Wax ester (C_37_ to C_40_)	*Chloroflexi* (genus *Roseiflexus*)	van der Meer et al., 2010
**Fatty acids**		
i*/a*-C_15_, *i/a*-C_17_	Gram-positive bacteria, sulfate-reducing bacteria	Taylor and Parkes, 1983; Lei et al., 2012
16:1 (ω5)	*Cyanobacteria*	Coates et al., 2014
16:1 (ω7)	*Cyanobacteria*	Ahlgren et al., 1992; Dijkman et al., 2010; Coates et al., 2014
Cy-C_17_	Gram-negative bacteria, anaerobic bacteria, sulfur- and iron-oxidizing bacteria, green sulfur bacteria	Kenyon and Gray, 1974; Jungblut et al., 2009
18:1 (ω7/9)	*Cyanobacteria*	Ahlgren et al., 1992; Dijkman et al., 2010; Coates et al., 2014
18:2 (ω6)	*Cyanobacteria*	Ahlgren et al., 1992; Jungblut et al., 2009; Dijkman et al., 2010
18:3 (ω6)	Microalgae/Diatoms/*Cyanobacteria*	Ahlgren et al., 1992; Jungblut et al., 2009; Dijkman et al., 2010
18:4 (ω3)	Microalgae/Diatoms/*Cyanobacteria*	Ahlgren et al., 1992; Dijkman et al., 2010
Cy-C_19_	Gram-negative bacteria, anaerobic bacteria, sulfur-oxidizing bacteria, purple sulfur bacteria	Grimalt et al., 1991; van der Meer et al., 1998; Jungblut et al., 2009
20:4 (ω10)	Diatoms	Ahlgren et al., 1992; Dijkman et al., 2010
20:5 (ω3)	Diatoms	Ahlgren et al., 1992; Dijkman et al., 2010

*For nomenclature details, see Figure 2.*

*^a^Most likely biological source, considering the compound-specific stable-carbon isotopic composition observed here (Figure 3).*

Low molecular weight compounds from 16 to 19 carbons [e.g*., n*-C_17_, C_17:1_, Me-C_17_, and DMe-C_17_, *n*-C_19_ alkanes; *n*-C_17_ alkanol; or 16:1 (ω5 or ω7), 18:1 (ω7 or ω9), and 18:3 (ω6) fatty acids] are often considered biomarkers, more or less, specific for cyanobacteria (see references in Table 4). For instance, prevailing peaks at *n*-C_17_ in molecular distributions of alkanes may be generally attributed to a cyanobacterial source in general, while the presence of heptadecane isomers or methyl heptadecanes (mono and di) may be more related to specific cyanobacterial clades (up to species), depending on the relative proportion of the isomers (Coates et al., 2014). In the present study, all the mentioned cyanobacterial biomarkers were observed to be dominant in the biofilms from temperatures ≤ 56°C (i.e., CW1, CE1, CE2, CE3, and CE4gr) (Figure 2), with also the presence of some of them [mostly the *n*-C_17_ and Me-C_17_ alkanes, as well as the 16:1 (ω7) fatty acid] in the highest-temperature biofilm (CE6). The latter, together with CE3 and CE4gr (and minorly CE5), showed a prevailing peak of heptadecane (*n*-C_17_), with a relatively large proportion of mid-chain methyl heptadecanes (Figure 2A), which resembles the hydrocarbon composition described for the cyanobacterial genus *Fischerella* (Coates et al., 2014; Teece et al., 2020). The presence of such an alkane pattern mostly in CE3 and CE4gr, but also in CE6 and, to a lesser extent, in CE5 (Figure 2A) is compatible with cyanobacterial communities dominated by *Fischerella* from temperatures ≥ 46°C. In contrast, the distinct hydrocarbon pattern in CW1, CE1, and CE2 (i.e., rather dominated by isomers of the C_17:1_ and/or *n*-C_15_; Figure 2A) pointed to the dominance of alternative genera of cyanobacteria.

The ubiquitous detection of phytol in the polar fraction of all biofilms revealed the generalized presence of phototrophs along the Cacao hydrothermal transect (Figure 2B). This alcohol is a degradation product of chlorophyll and may be produced by phototrophic organisms, including (mostly) cyanobacteria, green algae, and higher plants but also green sulfur and non-sulfur bacteria (Didyk et al., 1978). The relative abundance of phytol over other free alcohols may assist in discriminating the specific biological source based on its primary photopigment. For instance, the predominance of phytol over octadecanoic (*n-*C_18_) has been related to chlorophyll*a* and thus restricted to cyanobacteria (Shiea et al., 1991) or other oxygenic phototrophs (i.e., algae and higher plants), while the dominance of octadecanoic over phytol has been rather attributed to bacteriochlorophyll *c*_*s*_, the major pigment in anoxygenic photoautotrophs, such as *Chloroflexus aurantiacus* (Ward et al., 1989). In the Cacao biofilms, the prominent presence of phytol over octadecanoic in CW1, CE1, CE2, C3, and CE4gr was argued to be most likely related to cyanobacterial sources, as well as to eukaryotic phototrophs according to the detection of phytosterols, such as campesterol, stigmasterol, or β-sitosterol (Volkman, 2003) and polyunsaturated fatty acids [18:3(ω6), 18:4(ω3), 20:4, or 20:5(ω3); Ahlgren et al., 1992; Dijkman et al., 2010] mostly in CE1 and CE2 (Figures 2B,C). In contrast, the inverse predominance of octadecanoic over phytol in CE4, CE5, and CE6 rather pointed to relative enrichment in *Chloroflexus*-like microorganisms in the high-temperature biofilms.

The hypothesis of relative enrichment in green non-sulfur bacteria with temperature was supported by the detection of a series of HMW polyunsaturated alkanes from 29 to 32 carbons and a maximum peak at 31:3 (i.e., hentriacontatriene) in samples of temperature ≥ 46°C, mostly CE4, CE5, and CE6 (Figure 2A). These HMW polyunsaturated alkanes are described, together with wax esters from C_30_ to C_37_, as predominant lipidic compounds in mats of the thermophilic *Chloroflexus aurantiacus* and other phylogenetic relatives from the *Chloroflexi* phylum (van der Meer et al., 2008, 2000). In addition, wax esters from C_38_ to C_40_ have been also detected in *Chloroflexi* specimens, particularly from the species *Roseiflexus castenholzii* (van der Meer et al., 2010). In the Cacao stream, the detection of *Rosiflexus*-related wax esters was restricted to CE3 and CE4gr, whereas those associated with *Chloroflexus*-like microorganisms (from C_33_ to C_37_) were more ubiquitous from CE3 to CE5 (Supplementary Figure 4).

Other microbial sources inferred from the lipid biomarkers include sulfate-reducing bacteria (SRB), green sulfur bacteria (GSB), and purple sulfur bacteria (PSB). The presence of SRB has been largely related to the detection of *i*/*a*-pairs of the C_15_ and C_17_ fatty acids (Taylor and Parkes, 1983), whereas that of GSB or PSB has been rather associated with the presence of cyclo-propyl acids of either 17(Cy-C_17_) (Kenyon and Gray, 1974; Jungblut et al., 2009) or 19 (Cy-C_19_) carbons (Grimalt et al., 1991; van der Meer et al., 1998), respectively. In the Cacao stream, the mentioned lipid biomarkers were generally detected in all biofilms (Figure 2C), suggesting the ubiquitous presence of SRB, GSB, and PSB along the hydrothermal gradient, although with the greater relative abundance of SRB in CE2, CE3, and CE6; of GSB in CE4gr and CE4; and of PSB in CE6.

Finally, the contribution from eukaryotic sources other than green algae and higher plants was inferred from the detection of sterols, such as cholesterol and derivatives (i.e., present in animals or microalgae; Volkman, 2003), fucosterol (a biomarker of microalgae and macroscopic brown algae; Patterson, 1972; Mercer et al., 1974), and 5,6-dihydro ergosterol (likely derived from ergosterol, a biomarker of fungi; Patterson, 1972; Nes and McKean, 1977). Except for cholesterol and cholestanol, which were also detected in CE6, all other sterol compounds were almost exclusively found at temperatures ≤ 46°C, mostly in CE1 and CE2, which illustrates the preference of the local eukaryotic organisms (mostly those related to algae and plants) to low temperatures (≤32°C).

### Carbon Metabolism of Microbial Communities Along the Cacao Hydrothermal Transect

Compound-specific stable carbon isotope analysis may provide a rapid screening tool for carbon fixation pathways (Supplementary Text 1). Autotrophic organisms build their biomass by incorporation of inorganic carbon, primarily in the form of (mostly atmospheric) CO_2_ or dissolved inorganic carbon (mostly HCO_3_^–^), which show different carbon isotopic compositions (δ^13^C), varying from values as low as ca. –8‰ in atmospheric CO_2_ (Graven et al., 2017) to as high as from ca. –2 to 0‰ in bicarbonate (Mook et al., 1974). Because of the enzymatic preference for ^12^CO_2_ relative to ^13^CO_2_, carbon isotopic fractionation occurs during carbon fixation, a discrimination process against the heavy isotope that varies widely, depending on the carbon assimilation pathway (Hayes, 2001; Jahnke et al., 2001). Thus, knowing the bulk or compound-specific isotopic composition of a sample, we can infer the metabolic pathway followed by the autotrophs (Supplementary Text 1) and learn about the microbial community composition, as different phylogenetic lineages assimilate inorganic carbon typically, using a particular pathway (Hügler and Sievert, 2011).

In the Cacao stream, the compound-specific δ^13^C patterns of the lipids in three polarity fractions revealed different metabolic traits along the hydrothermal transect. In the apolar fraction, the compound-specific δ^13^C clustered into three groups of values (Figure 3A): (i) those highly enriched in ^13^C (i.e., δ^13^C from –12.3 to + 4.9‰) of the HMW compounds, (ii) those highly depleted in ^13^C (i.e., δ^13^C from –30.6 to –18.2‰) of the mid-molecular-weight compounds, and (iii) a much broader range of values, ranging from –29.9 to –8.4‰ of the LMW compounds. The most enriched δ^13^C values measured in the *Chloroflexus*-related HMW polyunsaturated compounds, mostly in CE4 and CE5 (Figure 2A), are compatible with the use of the 3HP bicycle (van der Meer et al., 2000). The most depleted δ^13^C values (i.e., ca. < -18‰) were detected in (linear or branched) saturated alkanes of all samples, but CE1 and CE2 (Figure 3A) were consistent with a Calvin-mediated carbon fixation (Hayes, 2001). Finally, the intermediate δ^13^C values (ca. from –18 to –8‰) largely found in CE1 and CE2 were, instead, compatible with a predominance of the rTCA pathway (Preuß et al., 1989; Hayes, 2001) or mixed participation of different metabolic routes. Typical users of the rTCA cycle that were detected in CE1 and CE2 are *Chlorobiales*, *Nitrospirae*, or ε-*Proteobacteria* (Takai et al., 2005; Hügler and Sievert, 2011). Thus, the compound-specific δ^13^C distribution in the apolar fraction reflects some fragmentation of the carbon assimilation pathways as a function of the temperature (Figure 3A), with a predominance of the rTCA at low temperatures, of the 3HP (mostly) from 56 to 67°C, and with the ubiquity of the Calvin cycle at all temperatures.

In the polar and acidic fractions, the distribution pattern of δ^13^C with the biofilm temperature was relatively less defined. Still, the range of δ^13^C values in the polar fraction (ca. from –26.7 to –2.3‰) covered fingerprints associable with the use of different carbon assimilation routes, with a general predominance of rTCA, together with 3HP in CE4 and CE5, or Calvin in CW1, CE3, and CE4gr (Figure 3B). In the acidic fraction, the range of δ^13^C values was even broader (ca. from –33.5 to +2.3‰) and covered even more carbon metabolic pathways. The compound-specific isotopic signature of the fatty acids in the low-temperature samples CE1 and CE2 reflected a predominant use of the rTCA pathway, while those in CE3 and CE4gr appeared to combine rTCA with the Calvin cycle. The most enriched δ^13^C values in CE4 and CE5 denoted instead relatively greater employment of the 3HP bicycle apart from the rTCA cycle, while the most depleted δ^13^C values in the highest-temperature CE6 suggested the use of the r-acetyl-CoA pathway, likely together with the Calvin cycle (Figure 3C). Interestingly, the most negative δ^13^C values in CE6 were detected in fatty acids of *i*/*a* configuration (*i/a*-C_15_, *i*-C_16_, *i/a*-C_17_, and *i*-C_18_) related to sulfate reduction (Taylor and Parkes, 1983; Jiang et al., 2012). SRB are metabolically versatile microorganisms that can degrade a wide variety of organic compounds heterotrophically, while some can also grow autotrophically, fixing inorganic CO_2_ into central metabolic intermediates like acetyl-CoA (Widdel and Hansen, 1992). The lowest δ^13^C values measured in CE6 could be explained by either the autotrophic assimilation of carbon through the r-acetyl-CoA pathway or the heterotrophic growth of SRB on isotopically depleted biomass (e.g., from r-acetyl-CoA or Calvin cycle users). The relevant contribution of the r-acetyl-CoA pathway in the high-temperature CE6 was reflected in the isotopic composition of the organic matter (i.e., TOC), which showed the lowest bulk δ^13^C value (Figure 3).

Considering together the stable-carbon isotopic composition of the lipid biomarkers from the apolar, polar, and acidic fractions, we conclude that the microbial transition along the hydrothermal transect is accompanied by an alternate of the carbon metabolic routes, where the use of the rTCA pathway is ubiquitous along the whole temperature spectrum, whereas the Calvin cycle is relatively more relevant mostly in samples up to 56°C, the 3HP bicycle in samples from 46 to 72°C, and the r-acetyl-CoA pathway almost exclusively in CE6 (i.e., 72°C).

### The DNA Sequencing Qualitatively Validated the Forensic Capacity of the Lipid Biomarkers to Identify Primary Biosources in the Cacao Hydrothermal Stream

The combination of molecular and isotopic analysis of lipid biomarkers allowed us to identify the primary biosources and autotrophic metabolism prevailing along the Cacao hydrothermal stream. Whereas a biological source assessment based on lipid biomarkers commonly entails a generalist reconstruction of the community structure, in this study, we were able to detect some relatively specific molecules (e.g., *Fischerella* related 4, 5, and 6 Me-C_17_ alkanes; *Chloroflexus-*attributed dominance of *n*-octadecanoic over phytol; or *Rosiflexus*-related C_38_ wax esters) that could be more precisely linked to particular biological sources (i.e., at the genus level). To test the validity of the identity assignation derived from using the lipid biomarkers, we first needed to learn about the phylogeny of the hydrothermal microbial community. To that end, we used a taxonomically specific technique such as DNA sequencing. We thus compared the microbial community structure built on 16S and 18S rRNA gene sequencing data, with the compositional reconstruction based on lipid biomarkers, to qualitatively calibrate the taxonomic diagnosis precision of the latter.

The DNA sequencing returned a microbial community structure that reflected relevant compositional differences between the samples. Overall, the microbial community was richer and more diverse in the low-temperature (≤32°C) samples (i.e., CW1, CE1, and CE2) than those above 46°C (Table 3), where the high temperature may favor the dominance of specialized microorganisms, resulting into less diverse communities. Interestingly, despite the low microbial richness and diversity measured in CE3 and CE4, these samples showed the highest biomass concentrations measured as TOC (Table 2). This could be related to a relatively large proportion of exopolysaccharides (i.e., a binding agent) in these slime and jelly-like biofilms at the steepest part of the transect compared to the other samples, situated in less dynamic zones and, instead, composed of thin layers, green flocculates, or disaggregated biomass of rougher and less jelly appearance. Furthermore, it cannot be ruled out that, considering the ever-changing conditions of hydrothermal systems, the actual temperature in this part of the hydrothermal channel is likely to move between 40 s °C and 50 s °C rather than being fixed at the putative 56°C measured at the time of sampling. Such a temperature range could be favorable/tolerable for both mesophilic and thermophilic microorganisms, thus leading to a relatively higher ecological productivity (i.e., biomass).

The compositional differences were already clearly visible at the phylum level (Figure 4A), where the microbial composition of the lowest-temperature biofilms (≤32°C) was largely dominated by *Proteobacteria* and *Cyanobacteria*, the mid-temperature biofilms (from 46 to 56°C) by *Cloroflexi* and *Cyanobacteria*, the high-temperature biofilms (from 56 to 67°C) by *Cloroflexi* and *Bacteroidetes*, and the highest-temperature biofilm (72°C) by *Deinococcus-Thermus, Chloroflexi*, and *Acetothermia*. The taxonomic differences observed along the thermal spectrum were generally mimicked at the isotopic level by the lipid biomarkers (Figure 3). Thus, the isotopic composition of the lipid biomarkers reflected a combined use of the rTCA and Calvin (mostly in CW1) cycles in samples with a high representation of *Proteobacteria* and *Cyanobacteria* (CW1, CE1, and CE2); a dominant use of the Calvin cycle in those with relatively higher abundance of *Cyanobacteria* (CE3 and CE4gr); a prevailing use of the 3HP bicycle in samples with the highest relative abundance of *Chloroflexi* (CE4 and CE5); and a combination of the Calvin and r-acetyl-CoA pathway in CE6, where the most representative phyla (*Deinococcus-Thermus* or *Acetothermia*) are thermophiles reported to fix inorganic carbon through the r-acetyl-CoA pathway (Takami et al., 2012; Badhai et al., 2015).

The taxonomic composition of the microbial community in the low-temperature samples (≤32°C) was largely composed of uncl. *Rhodobacteraceae*, uncl. γ-*Proteobacteria*, *Rubrimonas*, and uncl. *NB1-j* (Figure 4B). In contrast to CE1 and CE2 of fairly comparable *Proteobacteria* communities at the genus level, CW1 showed more relative abundance of uncl. α-*Proteobacteria*, uncl. γ-*Proteobacteria*, and uncl. *NB1-j.* While the set of lipid compounds detected did not include biomarkers specific enough to reflect the taxonomic composition of the Cacao *Proteobacteria* community, their compound-specific carbon isotopic composition provided instead metabolic clues that explained, to some extent, the taxonomic variability observed between the three samples. Overall, lipid compounds in CW1 were generally imprinted by Calvin δ^13^C signatures (Figure 3) consistent with their relative abundance of Calvin users, such as α-*Proteobacteria* and γ-*Proteobacteria* (Hügler and Sievert, 2011). In contrast, the higher δ^13^C values in CE1 and CE2 suggested relatively greater use of the rTCA cycle, potentially conducted by some of the abundant unclassified *Proteobacteria* (Figure 4B).

The relevant presence of *Cyanobacteria* in CW1, CE1, CE2, CE3, and CE4gr was correctly diagnosed by the lipidic approach, where several biomarkers of the mentioned phylum (*n*-C_15_, *n*-C_17_, C_17:1_, Me-C_17_ alkanes; and 16:1 and 18:1 fatty acids) were detected mostly ≤ 56°C (Figures 2A,C). Zooming in at the genus level, the DNA sequencing showed further compositional differences in the *Cyanobacteria* community, with *Rivularia* representing most of the cyanobacterial sequences in CW1; *Synechocystis* and uncl. *Nostocales* in CE1; uncl. *Oxyphotobacteria* and *Rivularia* in CE2; and *Fischerella* in CE3 and CE4gr (Figure 4C). The relevant presence of *Fischerella* from 46 to 56°C (>95% of the cyanobacterial sequences) was anticipated by the lipid biomarkers 5Me-C_17_ and 6Me-C_17_ (Coates et al., 2014; Teece et al., 2020), whose detection was prominent in CE3 and CE4gr (Figure 2A). In the biofilms from lower temperatures, the different distribution patterns of (apolar and acidic) cyanobacterial biomarkers (Figure 4) suggested an abundance of alternative genera. We hypothesize that the characteristic predominance of pentadecane (*n*-C_15_), together with certain isomers of heptadecene [17:1(II) and (IV)] and nonadecene [19:1 (I)] observed in CE3 (Figures 2A,C) could be related with a relative abundance of the class *Oxyphotobacteria* (Figure 4C), whereas the prevalence of 17:1 [II] among the isomers of heptadecene in CE1 (Figure 2A) could be related, instead, to the order *Nostocales* (Figure 4C). While a precise identification of the different isomers of heptadecene was not possible [i.e., 17:1(I-IV) in Figure 2A], each isomer could be related to specific cyanobacteria genera according to their different relative distribution in the samples CW1, CE1, and CE2 (Figure 4C).

In the *Chloroflexi* phylum, the analysis at the genus level of the samples, containing more than 1% of *Chloroflexi* sequences within the bacterial community, revealed compositional differences between samples (Figure 4D). From 46 to 56°C, the biofilms were largely composed of *Roseiflexus* (mostly CE3) and *Chloroflexus* (mostly CE4gr). From 56 to 67°C, the proportion of *Roseiflexus* decreased drastically at the expense of mostly *Chloroflexus*, whose abundance increased progressively with temperature. At 72°C, a completely different *Chloroflexi* community consisted greatly of *Thermoflexus* and minority proportions of *Chloroflexus* and *Thermomicrobium*. Overall, this *Chloroflexi* community structure agreed with the presence of a *Roseiflexus*-related C_38_ wax ester (van der Meer et al., 2010) only in CE3 and CE4gr (Supplementary Figure 4), and the ubiquitous detection of *Chloroflexus*-related biomarkers (HMW polyunsaturated alkanes and wax esters from C_33_ to C_37_ (van der Meer et al., 2000, 2008) in all samples from water temperature ≥ 46°C (Figure 2A and Supplementary Figure 5).

In sum, the taxonomic identification based on the DNA sequencing results confirmed, to a reasonable extent, the qualitative microbial community structure built on the lipid biomarkers. While the taxonomic specificity of the lipid biomarkers is not as high as that of the DNA due to their nature (i.e., lipids are structural components of cell membranes generally present in most phylogenetic groups), they can provide (more or less specific) both identity assignation and metabolic traits identification by combining their analysis at molecular and isotopic levels. Thus, the microbial community composition of the Cacao biofilms was defined, reaching in some cases relatively good taxonomic fidelity at phylum (e.g., *Cyanobacteria* or *Chloroflexi*) or even the genus (e.g., *Fischerella*, *Roseiflexus*, or *Chloroflexus*) level, whereas, in other cases, it responded to more general criteria, such as ecology or metabolism (e.g., thermophiles, SRB, GSB, or PSB). Therefore, using the DNA sequencing technique, we qualitatively validated the forensic capacity of the lipid biomarkers to reconstruct the microbial community in the Cacao hydrothermal stream and to trace the evolution of the biofacies with temperature.

### Ecological Singularities Along the Cacao Hydrothermal Stream

**FIGURE 5:**
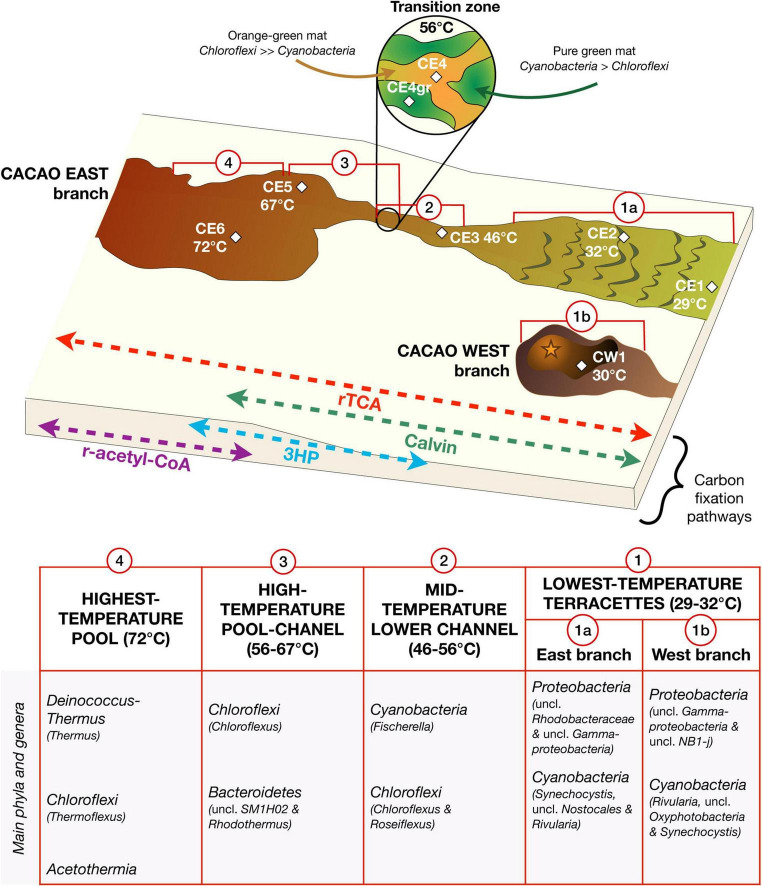
Ecological reconstruction of the microbial community distribution along the Cacao hydrothermal transect based on lipid biomarkers and DNA sequencing. The hydrothermal ecosystem was defined by a succession of microbial community structures and metabolic traits along the temperature gradient, with dominance of thermophilic species at high temperature, of anoxygenic photosynthetic species at intermediate temperatures (67–46°C), and mesophilic and oxygenic photosynthetic species at low temperature (≤32°C). In the channel, the compositional and metabolic transition at 56°C represented an ecological tipping point where aerobic and anaerobic conditions may alternate (from coexistence of *Cyanobacteria* and *Chloroflexi* to dominance of *Chloroflexi* and from a *Chloroflexi* community, largely photoheterotrophic to one mostly photoautotrophic). The abbreviation “uncl” means unclassified.

The combined (molecular and isotopic) analysis of lipid biomarkers with the DNA sequencing analysis allowed us to recreate the ecological system along the Cacao hydrothermal transect, where a transition of microbial components and prevailing metabolisms was observed from the hot hydrothermal vent to the cooler waters downstream (Figure 5). The prevalence of thermophiles at 72°C gave way to a microbial community dominated by *Chloroflexi* from 56 to 67°C, to one of the coexisting *Cyanobacteria* (mostly *Fischerella*) and *Chloroflexi* (*Roseiflexus* and *Chloroflexus*) from 46 to 56°C, and then, to a population of abundant mesophilic *Proteobacteria* (mainly *Rhodobacterales*) and *Cyanobacteria* (Figure 5). This microbial succession was coupled with a metabolic gradient from generally ubiquitous rTCA and prevailing r-acetyl-CoA and 3HP r-acetyl-CoA pathways at high temperatures toward Calvin assimilation routes as the water temperature decreased (Figure 5). The reconstruction of this sequence has ecological significance, as trends in the composition and metabolism of the microbiota along thermal gradients (from high to low temperature) somehow mimic the sequence of evolutionary events inferred by the SSU rRNA phylogenetic tree for the global biosphere, from thermophilic species to anoxygenic and oxygenic photosynthetic species (Farmer, 2000).

The thermophilic community at 72°C was largely composed of *Deinococcus-Thermus* (mostly *Thermus*), *Chloroflexi* (mostly *Thermoflexus*), and *Acetothermia* (Figure 4A), as well as *Crenarchaeota* (Supplementary Figure 5). The relative abundance of the orangish *Thermus* at the hottest site in Cacao (i.e., the hot spring vent at 72°C) agreed with the temperature distribution reported for the thermophilic genus in other hydrothermal systems from Island, the Yellowstone National Park, or the northern Thailand, where the temperature ranged from 75 to 89°C (Purcell et al., 2007). Other thermophilic components of CE6 were *Acetothermia*, a recently proposed phylum of thermophilic and chemolithotrophic bacteria likely related to *Thermotogae* (Rinke et al., 2013), and *Thermoflexus*, a heterotrophic bacterium from the *Chloroflexi* phylum (Thomas et al., 2021).

At 67°C, a first compositional shift was observed from dominant thermophiles to that of the *Chloroflexi* community (Figure 4D), with a prominence of *Chloroflexus* from 67 to 56°C (CE4) or *Roseiflexus* from 56 (CE4gr) to 46°C (CE3). The relative abundance of *Roseiflexus* or *Chloroflexus* was explained by the respective optimum growth of both genera from either 45 to 55°C and from 52 to 60°C, respectively (Pierson and Castenholz, 1974; Hanada et al., 2002). Their relative distribution in the Cacao hydrothermal stream was consistent with those previously described in hydrothermal systems from the Yellowstone National Park, such as the Mushroom and Octopus hot springs (Ward et al., 2012).

The compositional shift in the *Chloroflexi* community was accompanied by changes in the biofilm color (green-orange in CE4, intense green in CE4gr, and red-orange in CE3; Figure 1), which may, in turn, denote metabolic shifts. Whereas the red-orange-brownish tones of CE3 were associated with the relative abundance of *Roseiflexus* in this biofilm (Hanada et al., 2002), the green color in CE4gr was instead related to specific proportions of *Fischerella* and/or *Chloroflexus*. Concerning *Chloroflexus*-rich CE4, green and orange filaments in this biofilm could denote variable availability of oxygen, as *Chloroflexus* may form microbial mats of either dull green or orange aspect depending on aerobic or anaerobic conditions, respectively (Pierson and Castenholz, 1974). Thus, the coexistence of green and orange filaments in the stream middle channel suggests a possible metabolic transition in the *Chloroflexi* community at 56°C from photoheterotrophy to photoautotrophy. Species of both *Roseiflexus* and *Chloroflexus* are capable of growing photoheterotrophically by synthesis of chlorophyll in anaerobiosis, while some *Chloroflexus* sp. strains are photoautotrophic, fixing inorganic carbon through the 3HP bicycle (Pierson and Castenholz, 1974; van der Meer et al., 2010). While lipids synthesized through the 3HP bicycle are typically enriched in ^13^C (Hayes, 2001), those resulting from a heterotrophic growth are generally less enriched (i.e., more negative) because most of the carbon heterotrophically assimilated by the *Chloroflexaceae* derives from fermentation products and other photosynthates released by cyanobacteria (van der Meer et al., 2010) that fix carbon through the Calvin cycle (i.e., strongly depleted in ^13^C). In the Cacao stream, the slight isotopic depletion of the *Chloroflexi*-lipid biomarkers in the red-orange CE3 and the green CE4gr relative to those in CE4 and CE5 (Figure 3A) was compatible with a mixotrophic growth of the *Chloroflexi* community (mostly *Roseiflexus* in CE3, and mix of *Roseiflexus* and *Chloroflexus* in CE4gr) below 56°C, and photoautotrophic above 56°C (mostly *Chloroflexus* in CE4 and CE5). In CE3 and CE4gr, the co-occurrence of *Cyanobacteria* and *Chloroflexaceae* may explain a photoheterotrophic growth of the latter by cross-feeding on the relatively ^13^C-depleted cyanobacterial exudates (van der Meer et al., 2008). This would have resulted in isotopic compositions, ranging between those typical of strict use of either the Calvin or the 3HP cycle. In contrast, the negligible amount of *Cyanobacteria* in CE4 and CE5 (Figure 4A) would have limited the growth of the *Chloroflexus*-dominated *Chloroflexi* community to an autotrophic mode. Thus, the relative proportion of *Cyanobacteria* and *Chloroflexi* as determined by DNA sequencing appears to be a key to determine the isotopic signatures of the biofilms along the hydrothermal gradient, with 56°C representing a compositional and metabolic tipping point (Supplementary Figure 6).

A last compositional shift was observed at 46°C for a *Cyanobacteria* community dominantly composed of *Fischerella* to one represented by a variety of genera, such as *Synechocystis, Nostocales, Rivularia*, or *Oxyphotobacteria* at ≤ 32°C (Figure 4C). The detection of *Rivularia*, *Chroococcidiopsis*, *Cyanobacterium*, or *Nostocales* was consistent with previous descriptions of El Tatio (Phoenix et al., 2006; Gong et al., 2021; Wilmeth et al., 2021), whereas other genera, such as *Phormidium, Lyngbya, Leptolyngbya*, or *Calothrix*, previously identified in regional hot springs (Fernández-Turiel et al., 2005; Phoenix et al., 2006; Wilmeth et al., 2021), were not found in the present study. To the best of our knowledge, it is the first time that some genera of *Cyanobacteria* (e.g., *Synechocystis*, *Gloeocapsa*, or *Annamia*) are reported in the geysers field of El Tatio. The predominance of the genus *Fischerella* in the cyanobacterial community of Cacao from 46 to 56°C was comparable to that observed in a similar hydrothermal system at Yellowstone National Park (*White Creek*) between 39 and 55°C (Ward et al., 2012). However, the detection of *Fischerella* biomarkers occurs up to 72°C, a temperature very close to the upper limit of chlorophyll *an* (i.e., degradation at ∼73–74°C; Miller and Castenholz, 2000). While some species of *Fischerella* are considered to be thermophilic representatives of *Cyanobacteria* (i.e., able to grow well or best above 45°C; Castenholz, 1981), the maximum temperature at which this genus had been observed so far is ∼60°C (Ward et al., 2012; Wilmeth et al., 2021). While Wilmeth et al. (2021) have described recently the presence of *Fischerella* (11% of cyanobacterial sequences) in microbial mats at 61°C in hot springs from the Middle Basin at El Tatio, this is the first time that its biomarkers are found at a temperature as high as 72°C. We consistently detected in CE6 DNA sequences of *Fischerella* (901 sequences, representing 25% of the total cyanobacterial sequences in CE6; Supplementary Table 2), as well as lipid biomarkers associated with this genus (i.e., the relative abundance of 5Me-C_17_ and 6Me-C_17_; Coates et al., 2014; Figure 2A).

As a result, the microbial ecosystem in the Cacao hydrothermal stream was defined by a transition of microbial communities and metabolic traits along the temperature gradient, where 67, 56, and 46°C represented ecological tipping points. Understanding the principles of microbial community ecology in modern, operative thermal spring systems is paramount for learning how to interpret the paleobiology of ancient hydrothermal deposits on Earth.

### Paleobiological and Astrobiological Relevance of Understanding Modern Hydrothermal Ecosystems

Highly resistant lipid biomarkers offer a powerful forensic tool for the detection of biological signals over geologically relevant periods with relevance for paleobiology and astrobiology. In contrast to DNA and other labile biomolecules, lipid compounds can be preserved in the geological records for up to billions of years (Brocks et al., 2005) through mechanisms such as mineral-organic interaction, xero preservation, or mineral entombment (e.g., in silica sinter). Lipid profiles displaying comparable transitions with temperature to those observed here have been described in silica sinter deposits from worldwide hydrothermal systems; Yellowstone National Park in the US (e.g., Parenteau et al., 2014), the Taupo Volcanic Zone in New Zealand (e.g., Pancost et al., 2006; Campbell et al., 2015), or El Tatio in the Chilean *Altiplano* (e.g., Sánchez-García et al., 2019; Teece et al., 2020). To correctly underpin the paleoecological interpretation of fossil hot spring deposits, a detailed understanding of the primary biosignatures before silicification is essential. Here, we determined the molecular and isotopic fresh end members of the local microbiota at El Tatio to provide a kind of “qualitative calibration” of the lipid biomarkers at the local scale that will be a key to interprete fossil records in this or other similar spring systems on Earth. Furthermore, by unveiling the molecular and isotopic fingerprints of life in modern silica-rich hot springs with the same analytical technique (i.e., GC-MS) currently employed (a NASA-funded MSL SAM instrument) or to be employed soon (an ESA-funded ExoMars MOMA instrument), we contribute to provide robust criteria for recognizing hypothetical biogenic features in analogous silica deposits on Mars. The close resemblance of the nodular and digitate silica deposits at El Tatio with the opaline silica structures discovered by the Spirit rover at Gusev Crater makes the hydrothermal system of El Tatio a primary study site for deciphering fossil biomarkers records in ancient silica deposits on Mars. Interpreting its modern biosignatures in fresh biofilms is a key to advancing our strategies to explore signatures of life or prebiotic chemistry on silica structures on Mars.

However, the interpretation of potential life signals beyond the Earth requires extreme caution, especially regarding the possible ambiguity of biogenicity. The Astrobiology Community agrees that the first point to elucidate when dealing with possible extraterrestrial biosignatures is to discern the actual biogenicity of the signal to avoid “false positives” (i.e., morphologies, textures, patterns, or compounds that resemble life but are abiotic). There are several ways to differentiate abiotically synthesized from biologically generated lipid compounds. One criterion for discriminating between biogenic vs abiogenic origin of organics is attending to the molecular distribution within a compound family or polarity fraction (i.e., hydrocarbons, fatty acids, or alcohols). Abiotic hydrocarbons resulting from Fischer-Tropsch reactions show typical Schultz-Flory distributions of smooth, homogeneous decreasing abundance with increasing carbon numbers (Szatmari, 1989), absence of molecular patterns, and lack of even-over-odd or odd-over-even carbon number predominance in oxygenated (fatty acids and alcohols) or defunctionalized hydrocarbon (alkanes and alkenes) series, respectively (McCollom et al., 1999). In contrast, biogenic lipid series, instead, show irregular distributions with a preference for the even or odd carbon numbers, the presence of one or more peaks of compounds standing out from the rest (e.g., in this study, cyanobacterial heptadecane and heptadecane isomers or *Chloroflexus*-associated long-chain polyunsaturated alkenes in the apolar fraction; Supplementary Figure 3A), as well as of other compounds than saturated and *normal* hydrocarbons (e.g., isoprenoids, monomethyl alkanes, or hopanes). In addition, a Schultz-Flory distribution of synthetic hydrocarbon mixtures produces nearly constant ratios (below ∼0.6) of the compounds with successive carbon numbers (i.e., C_*n+*1_/C_*n*_; Szatmari, 1989), while biogenic alkane series instead generate variable and heterogeneous values of the ratio.

Another criterion often used to assess the biogenicity of a carbonaceous material is considering its stable carbon isotopic composition. Overall, ^13^C-depleted signatures derived from a strong fractionation against the heavier carbon isotope relative to the inorganic source of carbon are typically attributed to biological sources, since no known inorganic process results in long-chain carbonaceous material being depleted in ^13^C as living organic matter (Peters et al., 2005). However, laboratory experiments reproducing Fischer-Tropsch reactions have reported synthesized hydrocarbons with δ^13^C values as depleted relative to CO_2_ as much as 36‰ (McCollom and Seewald, 2006; McCollom et al., 2010). Still, when analyzing intramolecular carbon atoms within larger lipid compounds, a more robust biosignature can be achieved (e.g., different δ^13^C values in alternating carbons, such as those observed in this study; Figure 3). Fischer-Tropsch synthesized organic compounds typically have a constant ^13^C composition for all carbons (McCollom and Seewald, 2006), whereas fatty acids and other compounds formed by biosynthetic pathways usually have distinctive ^13^C signatures between alternating compounds (i.e., even- versus odd-numbered carbon atoms within a series of *normal* lipids), arising from the fatty acid assembly mechanism (i.e., the addition of 2-carbon acetyl units, where the methyl and carboxyl groups differ substantially in their isotopic composition). While it may be difficult to differentiate biogenic from abiotic organic compounds based solely on the isotopic composition of bulk biomass or even individual compounds, the combination of the isotopic analysis of intramolecular carbon atoms with a rigorous analysis at a molecular level brings together the identification of not only one but several individual biomarkers may help to overcome the risk of assigning a biological origin to purely abiotic compounds. Thus, the risk of pitfalls and false positives will be minimized with the increasing complexity of the biosignature, so a co-occurrence of numerous anomalies or patterns in the overall molecular profile of extraterrestrial organic matter could still be strongly suggestive of biosynthesis (Marshall et al., 2017; McMahon and Cosmidis, 2021).

In this study, the extensive set of diverse and relatively complex lipids with variable but generally depleted carbon isotopic signatures of heterogeneous distribution within homologous lipid series, together, attest to a biogenic fingerprint. In the same geyser field of El Tatio, sinter deposits are reported to display bulk δ^13^C values comparable to those measured here for the fresh biofilms, more similar to CE3 and CE4 in proximal slopes (40–75°C; from –9.9 to –16.9‰), or to CE6 in near vent deposits (> 75°C; from –15.9 to –25.1‰) (Muñoz-Saez et al., 2020). More specifically, individual δ^13^C values of apolar (from –22 to –28‰), acid (from –20 to –37‰), and polar (from –25 to –30‰) lipids contained in sinter deposits from hydrothermally active and inactive geyser mounds in El Tatio (Sánchez-García et al., 2019) were also in the (lower) range of those measured here in the eight fresh biofilms. By recording the fresh biosignatures in the active hydrothermal system of Cacao, we dispense a molecular and isotopic template for the recognition of past biosignals in silicified counterparts on Earth and (if any) on Mars in analogous silica-rich hydrothermal systems. By spanning a wide gradient of temperature, we increase our chance to capture a broader variety of microorganisms and metabolisms and, so, a more comprehensive variety of lipid profiles. According to the hypothesis of possible extraterrestrial life, “as we know it” by Bartlett and Wong (2020), lipids emerge as ideal target compounds with a double astrobiological value, as potential biomarkers of universal (ubiquity in all types of organisms as components of their cell membranes) and paleo-reconstructive (high potential for preservation in the fossil record) character.

## Conclusion

This study aimed to interpret biosignatures in modern hydrothermal systems to learn how to decipher fossil records in ancient silica deposits. Exploiting the diagnosis potential of highly resistant lipid biomarkers, we characterized the primary biological sources and prevailing carbon metabolic routes operating in the modern hydrothermal ecosystem. The taxonomic precision (at the phylum and even at the genus level sometimes) of the lipid biomarkers-diagnosis tool was validated by parallel DNA sequencing, and its forensic capacity to identify primary biosources and autotrophic metabolisms calibrated at the local scale.

Together, the molecular, isotopic, and genetic results provided a global understanding of the modern ecosystem at the Cacao site and revealed a compositional and metabolic succession along the thermal gradient, from a majority of thermophiles (*Deinococcus-Thermus*, *Chloroflexi*, and *Acetothermia*), growing mostly by assimilation of inorganic carbon through the r-acetyl-CoA pathway at 72°C; to dominance of anoxygenic photosynthetic species (mostly *Chloroflexus*), primarily using the 3HP bicycle to grow from 56 to 67°C; to coexistence of anoxygenic (*Roseiflexus* and *Chloroflexus*) and oxygenic (*Fischerella*) photosynthetic species that grow photoheterotrophically (crossfeed on cyanobacterial exudates) or photoautotrophically (the Calvin cycle), respectively, from 56 to 46°C; and to eventual dominance of mesophilic oxygenic phototrophs from *Proteobacteria* (mostly *Rhodobacterales*) and *Cyanobacteria* (*Synechocystis, Nostocales, Rivularia*, or *Oxyphotobacteria*) that combined the use of the rTCA and Calvin cycles at temperatures of ≤ 32°C.

The detection of several singular ecological traits along the Cacao stream (e.g., presence of *Fischerella* up to 72°C; first identification of the cyanobacterial genera *Synechocystis* at El Tatio, *Gloeocapsa*, or *Annamia*; or the likely alternate at 56°C of aerobic and anaerobic conditions, with a transition from photoheterotrophic to photoautotrophic growth of the *Chloroflexi* community) illustrated the ecological uniqueness of the Cacao stream and highlighted the astrobiological interest of El Tatio. The hydrothermal system and its deposits provide excellent settings to advance the understanding of the limits of life and the emergence of early life on Earth. The taxonomically calibrated microbial community structure built on the lipid biomarkers provided a local (molecular and isotopic) fresh end member to recognize past biosources and metabolisms from altered biomarkers profiles in ancient silica deposits. Correlating resistant lipid biomarkers with ancestral metabolisms is critical for interpreting molecular biomarkers in analog planetary contexts.

## Data Availability Statement

Raw DNA sequence reads were deposited at the NCBI Sequence Read Archive (SRA) under the BioProject ID PRJNA777431 (https://www.ncbi.nlm.nih.gov/bioproject/PRJNA777431).

## Author Contributions

LS-G designed the present case study and collected the samples together with VP and NC. VM and LS-G conducted the extraction and molecular analysis of the lipid biomarkers, while DC took care of the stable-carbon isotopic analysis. VM and ML were in charge of extracting the DNA and analyzing and interpreting the sequencing data. VM and LS-G interpreted the lipid biomarkers results, integrated all the results, and wrote the manuscript, with the contribution of all co-authors. All authors contributed to the article and approved the submitted version.

## Conflict of Interest

The authors declare that the research was conducted in the absence of any commercial or financial relationships that could be construed as a potential conflict of interest.

## Publisher’s Note

All claims expressed in this article are solely those of the authors and do not necessarily represent those of their affiliated organizations, or those of the publisher, the editors and the reviewers. Any product that may be evaluated in this article, or claim that may be made by its manufacturer, is not guaranteed or endorsed by the publisher.
