# Supplementary material for: Lipid Profiles From Fresh Biofilms Along a Temperature Gradient on a Hydrothermal Stream at El Tatio (Chilean Andes), as a Proxy for the Interpretation of Past and Present Biomarkers Beyond Earth
Source: Front Microbiol. 2022 Jun 27;13:811904. doi: 10.3389/fmicb.2022.811904 (PMC9271869; doi:10.3389/fmicb.2022.811904)
Supplement: Supplementary file 1 [file Table_1.DOCX]

Supplementary Material

# Supplementary Data

**Text S1:** Carbon fixation pathways based on stable-carbon isotopic composition.

There are four primary carbon fixation pathways, which apply different degrees of fractionation that result into organic matter more or less depleted in ^13^C respect to the carbon substrate. 1) The Calvin-Benson-Bassham or Calvin cycle used by oxygenic photosynthetic organisms such as cyanobacteria, algae, or plants fixes CO_2_ with typical fractionations from 10‰ to 22‰ that yield biomass with δ^13^C values from ca. -18‰ to -30‰ (Hayes, 2001). A smaller fractionation (from 2‰ to 13‰ relative to their inorganic carbon source) is applied by anoxygenic phototrophs that assimilate CO_2_ or HCO_3_^-^ by using either the 2) reductive tricarboxylic acid (rTCA) cycle (e.g., *Chlorobiales*, *Aquificales*, *Nitrospirae*, or ε-*Proteobacteria*), or the 3) 3-hydroxypropionate (3HP) bicycle (*Chloroflexiaceae*), respectively. Accordingly, the biomass resulting from using the rTCA pathway typically has δ^13^C values from -12‰ to -21‰ (Preuss et al., 1989), while that derived from the 3HP bicycle values, from -4‰ to -15‰ (van der Meer et al. 2000). Finally, 4) the reductive acetyl-coenzyme A (r-acetyl-CoA) pathway (a.k.a. the Wood-Ljungdahl pathway) causes the greatest fractionation (from 20‰ to 36‰ relative to CO_2_) that result in the most ^13^C-depleted biomass (δ^13^C from -28‰ to -44‰) (Preuss et al., 1989). In turns, the synthesized lipids are usually even more depleted than the bulk cell material (*i.e*., mixture of isotopically heterogeneous compound classes) in all pathways except the rTCA cycle (Van der Meer et al., 1998; Jahnke et al., 2001) and sometimes the 3HP bicycle (Van der Meer et al., 2008).

**Text S2:** PCR and sequencing protocol realized by *Fundación Parque Científico de Madrid*

DNA extractions were first quantified with Picogreen (Invitrogen) and then 3 ng were used as input in a first PCR using a Q5 Hot Start High-fidelity DNA Polymerase kit (New England Biolabs, Massachusetts, USA). PCRs were performed with 22 cycles for all amplicons except for the bacterial 16S rRNA gene, which was performed with 20 cycles. A second PCR with 13 cycles was performed for all amplicons with the Q5 Hot Start High-fidelity DNA Polymerase kit to insert sample-specific barcodes and the Illumina adapter sequences (*i.e.,* 5’-AATGATACGGCGACCACCGAGAT CTACACTGACGACATGGTTCTACA-3' and 5'- CAAGCAGAAGACGGCATACGAGAT-[10 nucleotides barcode]-TACGGTAGCAGAGACTT GGTCT-3') (Fluidigm, San Francisco, CA, USA). Final amplicons were validated and quantified with a 2100 Bioanalyzer (Agilent, Santa Clara, CA, USA) and equimolar pools were purified by agarose gel electrophoresis in the case of the 18S rRNA gene pool, or AMPure Beads (Beckman Coulter, Pasadena, USA) in the case of the 16S rRNA gene of bacteria, cyanobacteria and archaea. The negative control showed absence of genes amplification and then was removed for downstream analysis. Amplicons were titrated by quantitative PCR using the Kapa-SYBR FAST qPCR kit for LightCycler480 (Merck KGaA, Darmstadt, Germany) and a reference standard for quantification. Final amplicon pools were denatured before seeding on a flowcell and sequenced using the MiSeq Reagent kit v3 (Illumina Inc. San Diego, CA) in a 2x300 pair-end sequencing run on an Illumina MiSeq sequencer (Illumina Inc.).

# Supplementary Tables

# Table S1: Excel file (separate) with the concentration (mg·g^-1^ of dry weight) of the different lipid compounds identified in the eight Cacao biofilms in three polarity fractions; (Tab 1) apolar, (Tab 2) polar, and (Tab 3) acidic.

**Table S2:** Relative abundance of *Cyanobacteria* genera among the eight Cacao stream biofilms based on the high-throughput sequencing of the cyanobacterial specific 16S rRNA gene.

|  | **Relative abundance (%)** | | | | | | | |
| --- | --- | --- | --- | --- | --- | --- | --- | --- |
| **Genera** | **CW1** | **CE1** | **CE2** | **CE3** | **CE4gr** | **CE4** | **CE5** | **CE6** |
| *Annamia* | 0.00 | 3.30 | 0.16 | 0.00 | 0.00 | 0.00 | 0.00 | 0.00 |
| *Arthrospira* | 0.00 | 0.01 | 0.00 | 0.04 | 2.00 | 0.00 | 2.80 | 0.00 |
| *Chroococcidiopsis* | 0.00 | 1.43 | 2.59 | 0.00 | 0.00 | 0.00 | 0.00 | 1.40 |
| *Cyanobacteriaceae* | 0.23 | 0.60 | 1.23 | 0.00 | 0.00 | 0.02 | 14.95 | 13.42 |
| *Cyanobacterium* | 0.01 | 0.03 | 0.01 | 1.82 | 0.04 | 0.00 | 0.00 | 0.00 |
| *Dactylococcopsis* | 0.00 | 0.00 | 0.00 | 0.00 | 0.00 | 0.00 | 0.00 | 1.71 |
| *Fischerella* | 0.00 | 1.01 | 4.69 | 97.99 | 95.95 | 99.94 | 46.73 | 25.20 |
| *Gleocapsa* | 0.00 | 10.29 | 1.92 | 0.00 | 0.00 | 0.00 | 4.67 | 0.31 |
| *Gloeocapsa* | 0.00 | 0.00 | 0.04 | 0.00 | 0.02 | 0.00 | 28.04 | 1.17 |
| Unclassified *Nostocales* | 0.00 | 29.35 | 0.75 | 0.00 | 0.00 | 0.00 | 0.00 | 0.48 |
| *Oscillatoria* | 0.00 | 0.02 | 2.19 | 0.00 | 0.00 | 0.00 | 0.00 | 1.96 |
| Unclassified *Oxyphotobacteria* | 10.32 | 2.70 | 30.76 | 0.03 | 0.34 | 0.00 | 0.93 | 15.72 |
| Unclassfied *Phormidesmiales* | 0.16 | 0.01 | 0.00 | 0.11 | 1.64 | 0.02 | 0.00 | 1.09 |
| *Rivularia* | 75.62 | 0.86 | 40.75 | 0.00 | 0.01 | 0.02 | 1.87 | 19.88 |
| *Synechocystis* | 8.10 | 44.73 | 14.39 | 0.00 | 0.00 | 0.00 | 0.00 | 7.94 |
| Unclassified *Eurycoccales* | 4.36 | 0.04 | 0.00 | 0.00 | 0.00 | 0.00 | 0.00 | 0.00 |
| Unclassified *Nostocales* | 0.00 | 4.94 | 0.23 | 0.00 | 0.00 | 0.00 | 0.00 | 7.69 |
| Unclassified *Pseudanabaenales* | 1.01 | 0.01 | 0.00 | 0.00 | 0.00 | 0.00 | 0.00 | 0.00 |
| Rest of the genera | 0.67 | 0.28 | 0.00 | 0.00 | 0.00 | 0.39 | 0.20 | 0.01 |

# Supplementary Figures


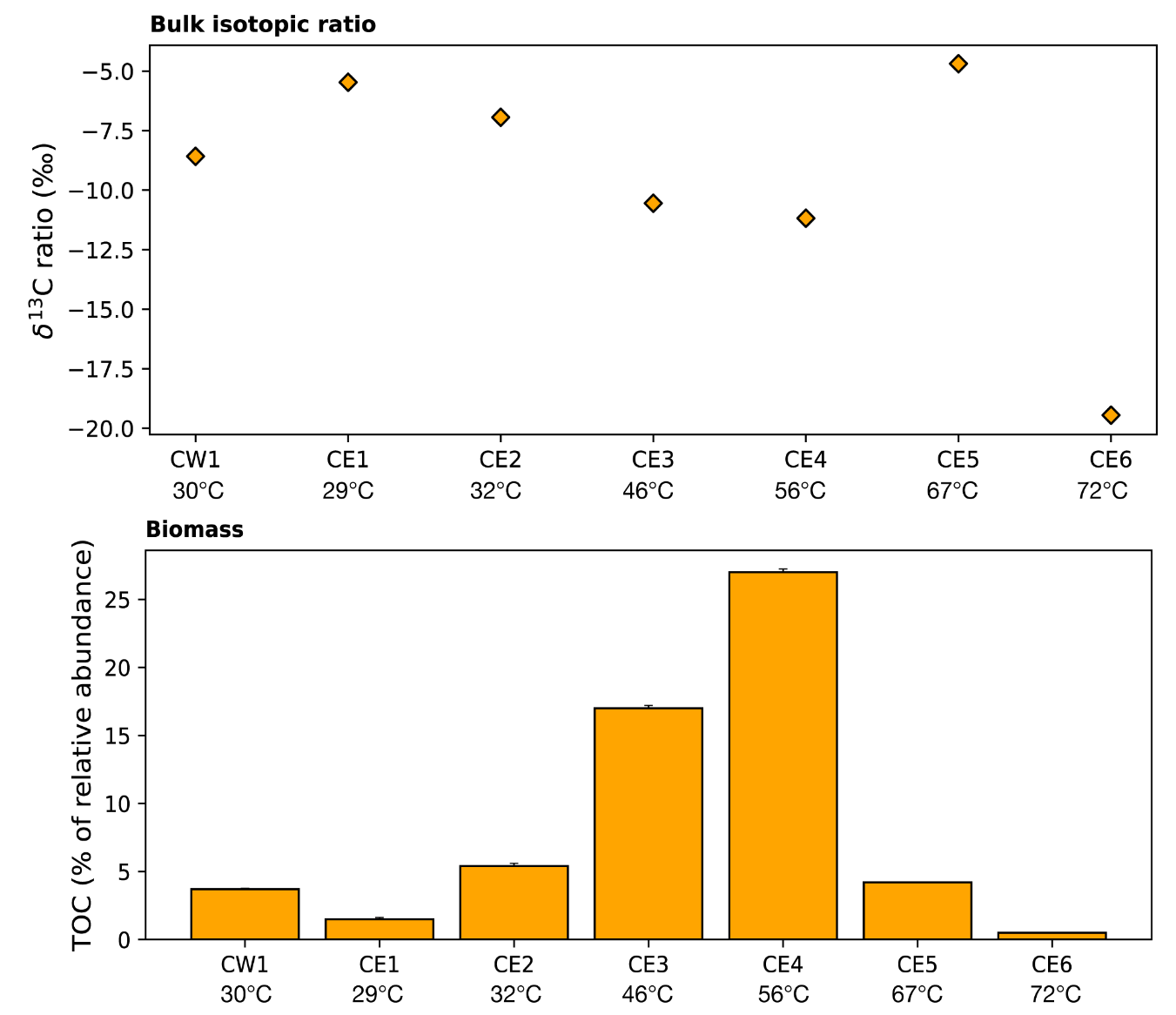


**A**

**B**

**Figure S1: (A)** Bulk geochemical characterization of the eight biofilms collected from the West (CW) and East (CE) branches of the Cacao hydrothermal stream. **(B)** Stable carbon isotopic composition (δ^13^C) of the total organic carbon (TOC).


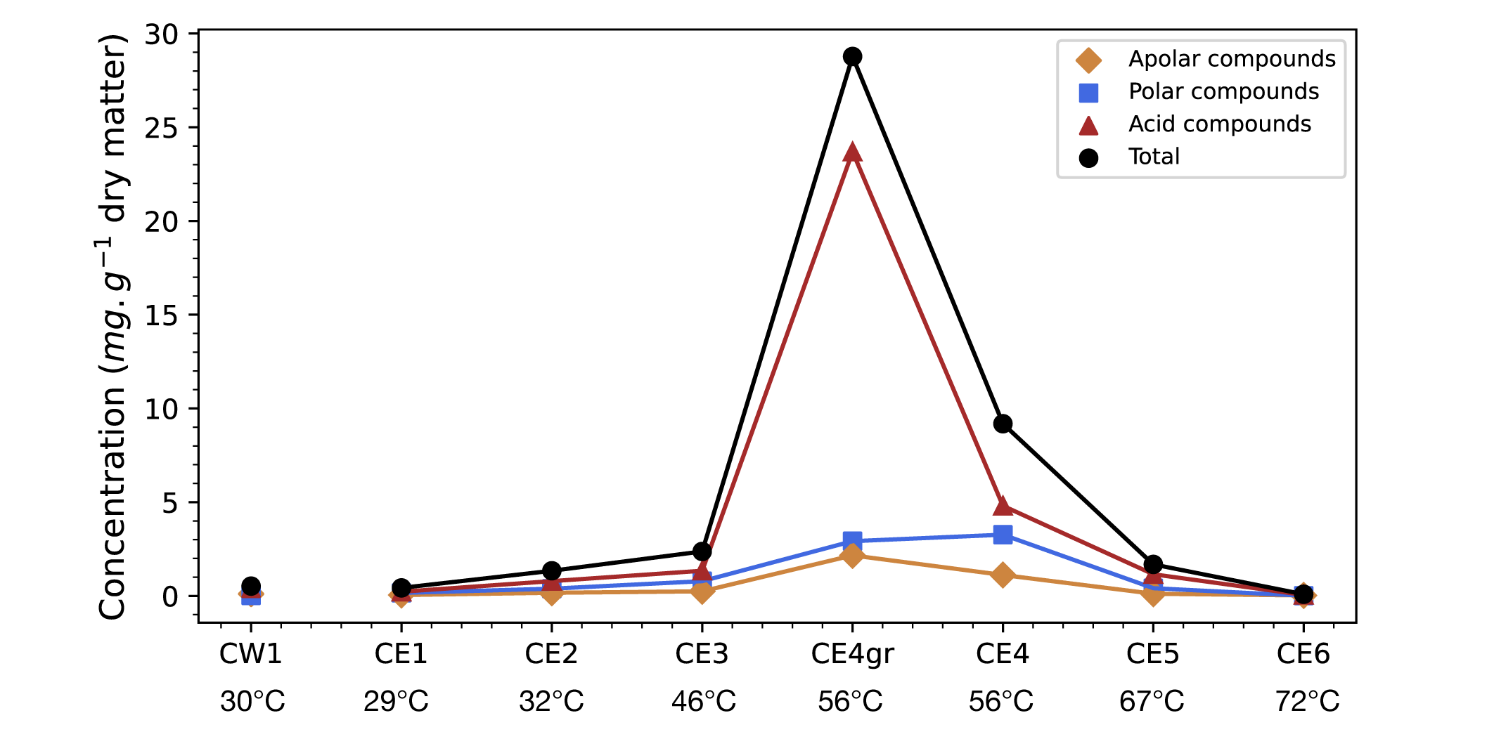


**Figure S2:** Total concentration of lipid compounds identified in three polarity fractions extracted with organic solvents from the eight biofilms from the West (n=1) and East (n=7) branches along the Cacao hydrothermal transect.


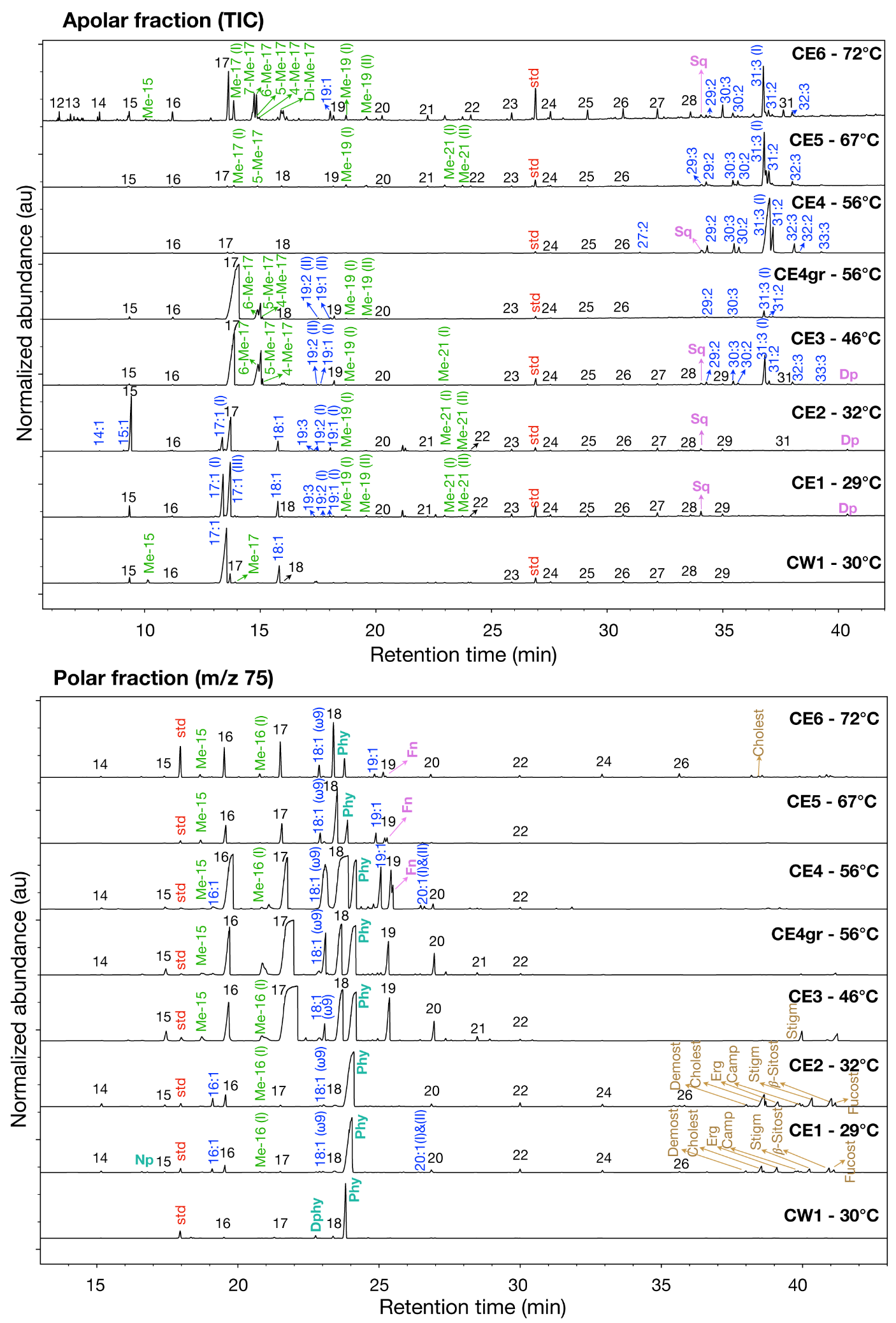


**A**

**B**


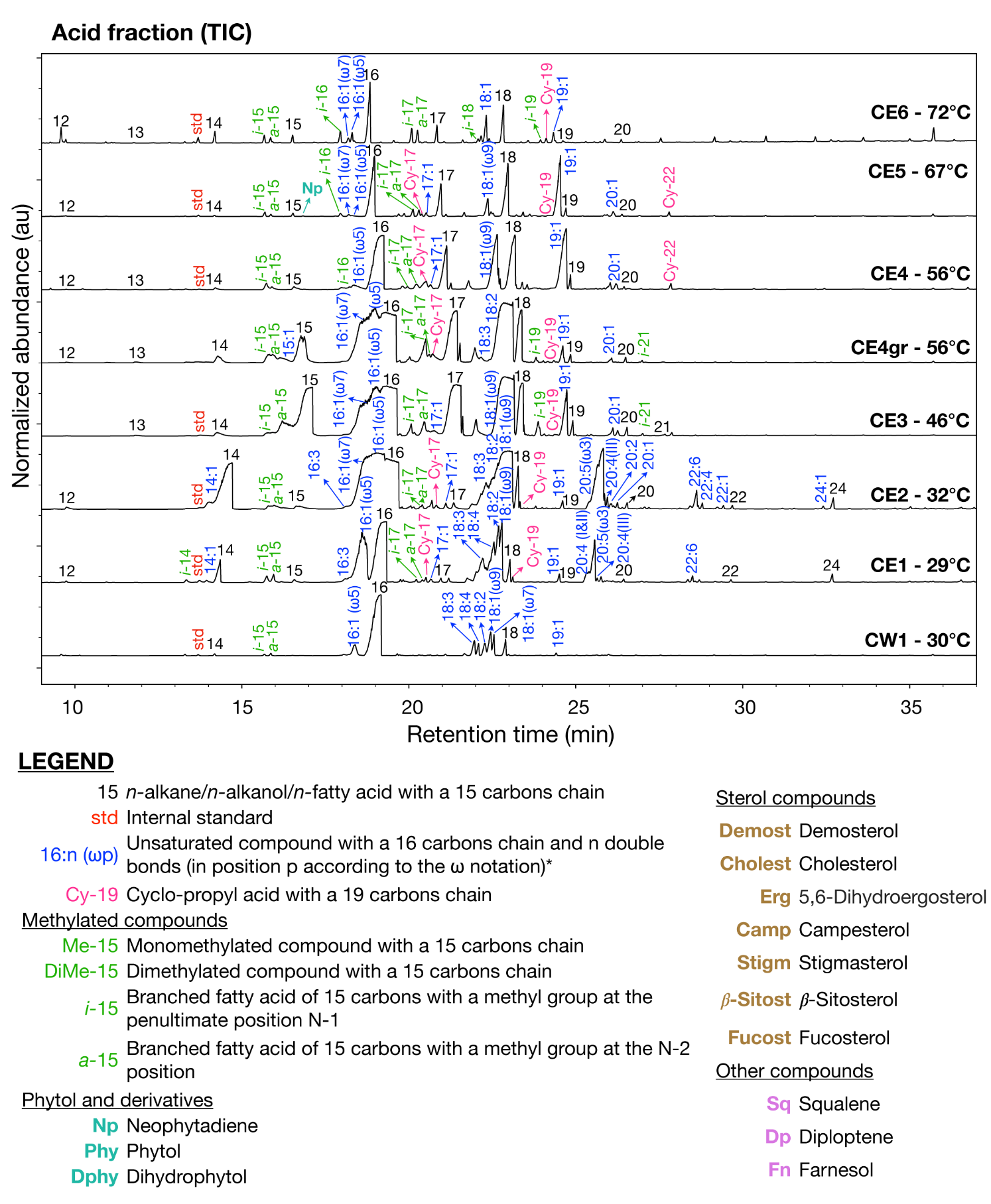


**C**

**Figure S3:** Mass chromatograms of the three polarity fractions extracted from the eight Cacao biofilms with identification of the most abundant lipid compounds. All the chromatograms are normalized to their maximum signal for clarity. **(A)** Chromatograms of the apolar fraction represented as total ion current (TIC; *i.e*. summed intensity across the entire range of masses), with identification of linear and saturated (*normal*) chains (*n*-alkanes), branched (methylated) and unsaturated (chains with double bonds) hydrocarbons. **(B)** Partial *m/z* 75 mass chromatograms of the polar fraction with identification of *n*-alkanols, sterols and other polar compounds (methylated and unsaturated alkanols). Only the ion 75 has been represented to remove alkanes compounds that were partially (but minorly) extracted in the polar fraction. **(C)** TIC chromatograms of the acidic fraction with identification of *normal* (*n*-fatty acids), branched, and unsaturated alkanoic acids. All the identified compounds and their corresponding concentration is displayed in the supplementary data excel file.

**
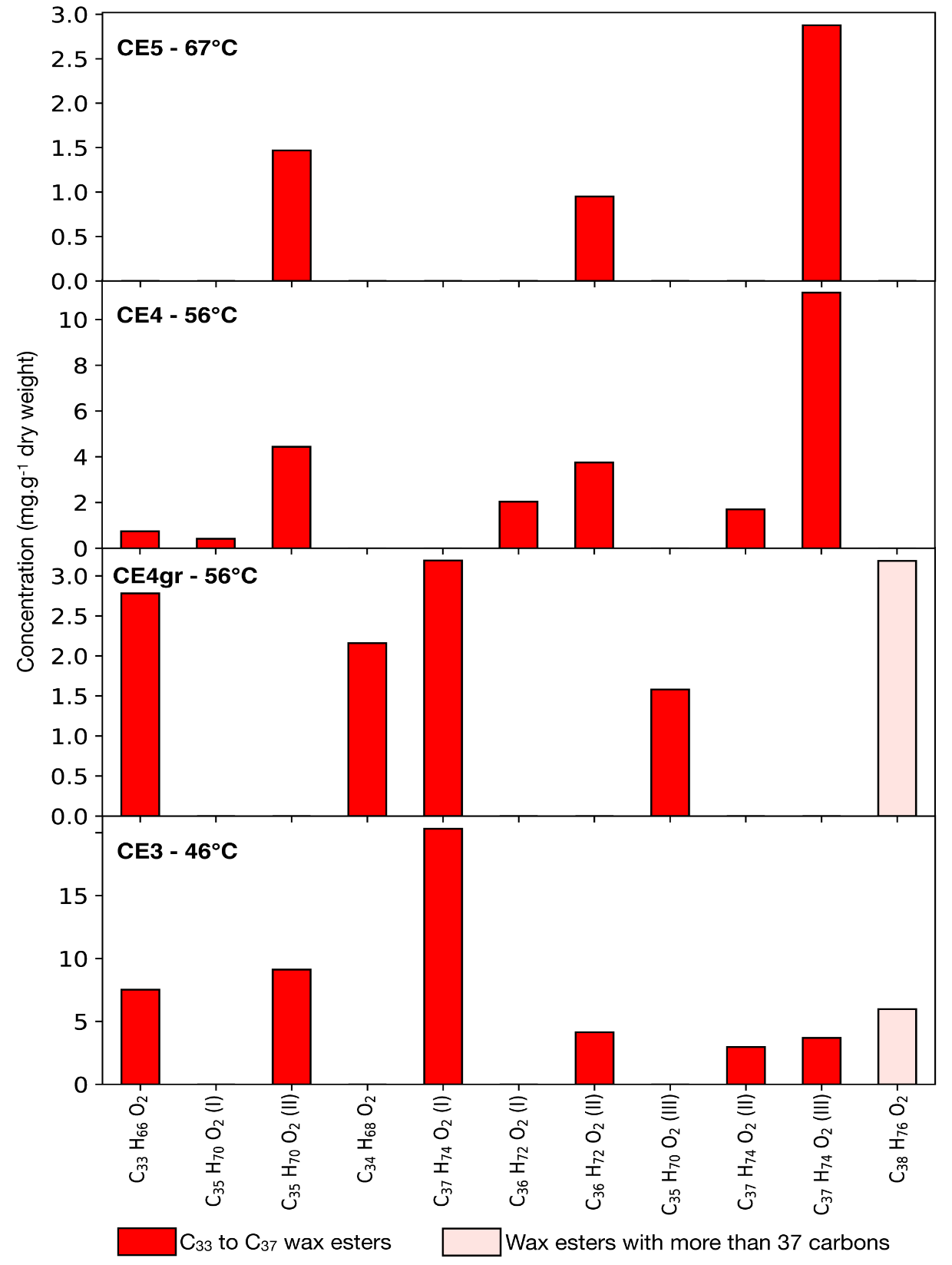
**

**Figure S4:** Concentration (mg·g^-1^ of dry weight) of the different wax ester compounds identified in the biofilms from temperatures between 46°C and 67°C. Wax esters with more than 37 carbons were represented in a different color (yellow) as they are considered as specific lipid biomarkers of *Roseiflexus castenholzii* (van der Meer et al. 2010).


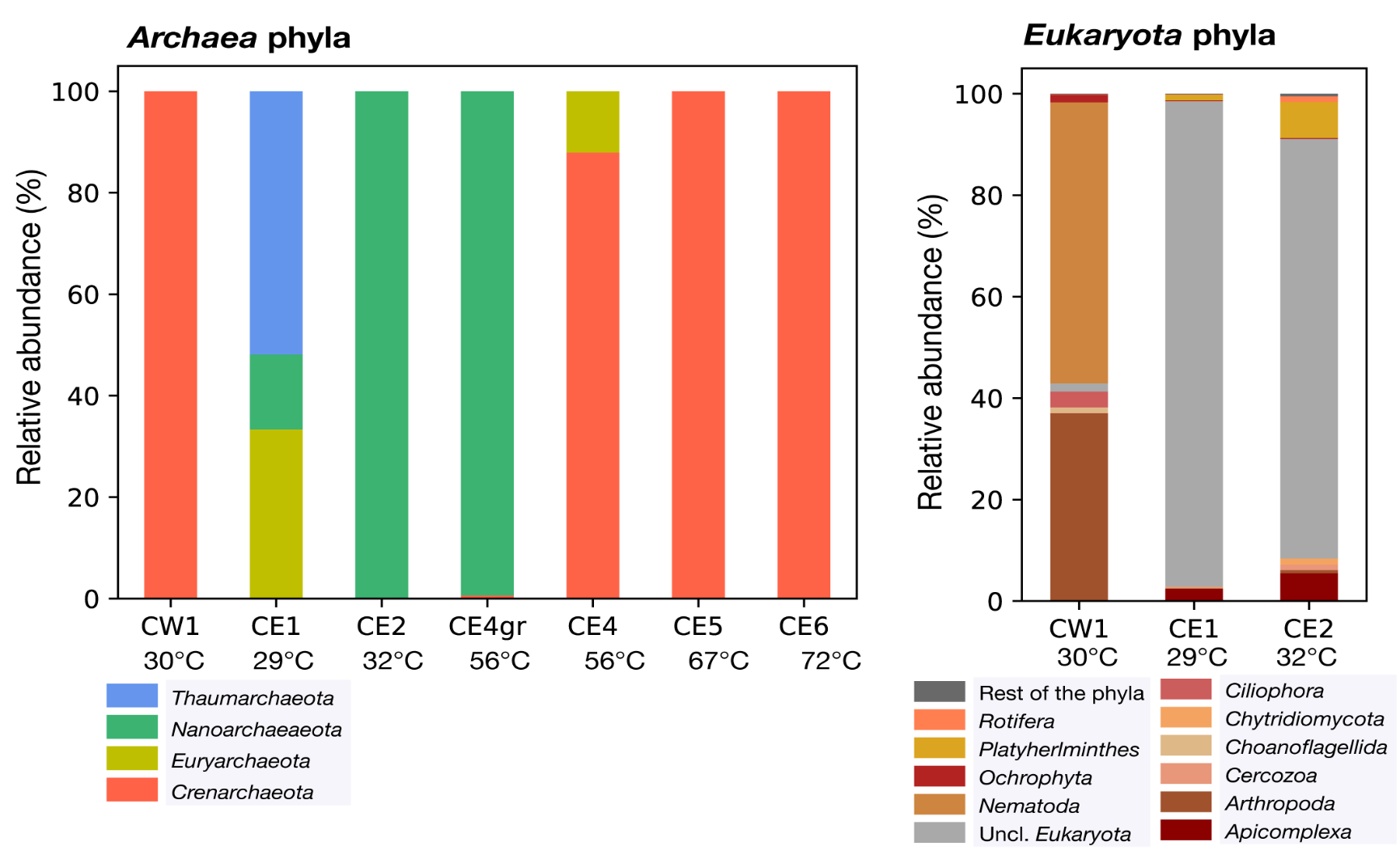


**A**

**B**

**Figure S5:** Composition of the *Archaea* **(A)** and *Eukaryota* **(B)** communities in the Cacao stream biofilms inferred from the 16S and 18S rRNA gene sequencing, respectively. **(A)** Relative abundance of archaeal sequences in all biofilms but CE3 that gave absence of archaeal sequences after quality-filtering (46°C). **(B)** Relative abundance of the eukaryotic sequences in the low-temperature biofilms (CW1, CE1 and CE2) where the 18S rRNA gene could be amplified. The abbreviation “uncl” refers to unclassified phyla.

**
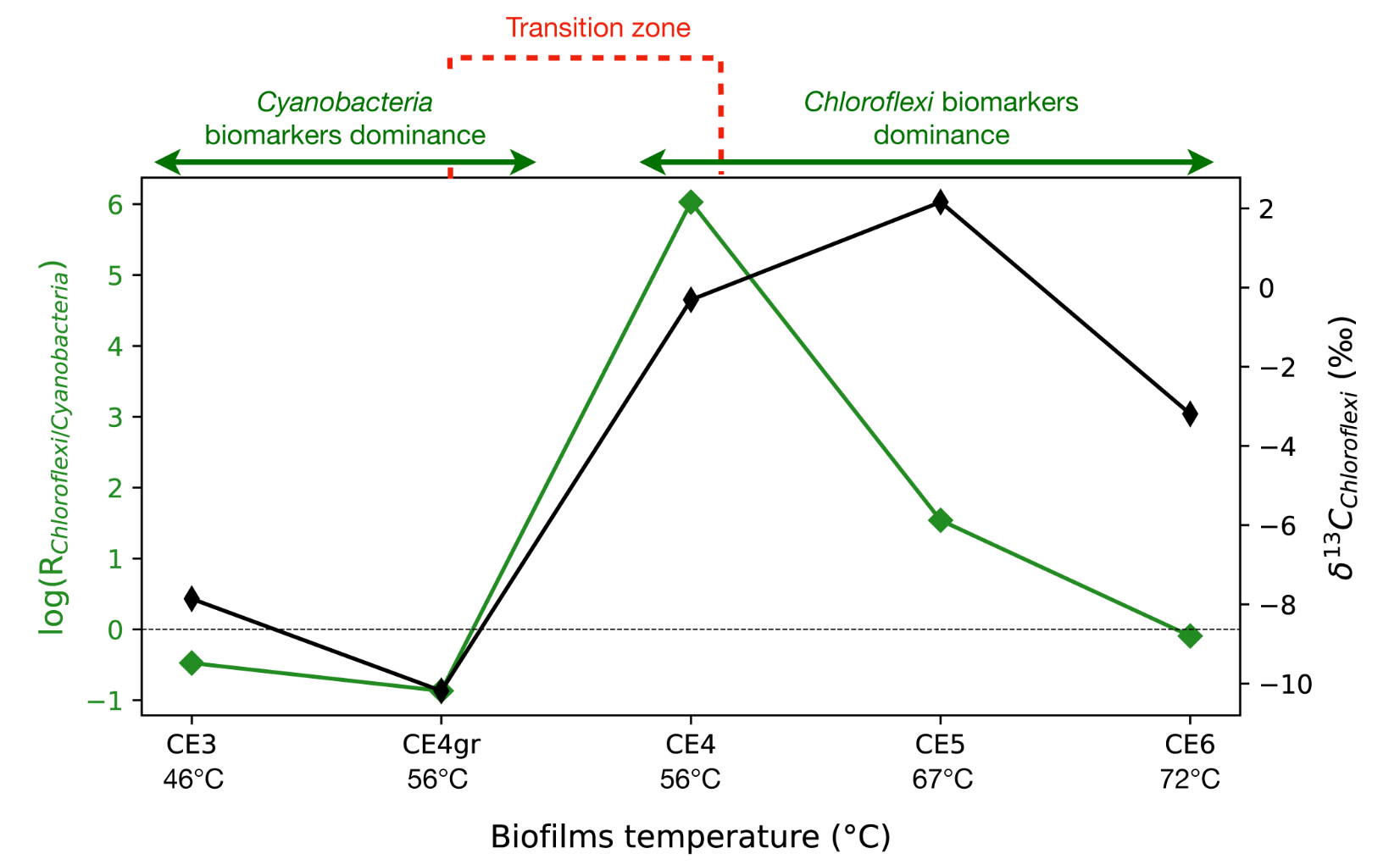
**

**Figure S6:** Relative distribution of *Cyanobacteria* versus *Chloroflexi* lipid biomarkers and isotopic composition of *Chloroflexi* lipid biomarkers in the high-temperature biofilms. In green, logarithm of the ratio of specific biomarkers of *Chloroflexi* (sum of long-chain polyunsaturated alkanes) over *Cyanobacteria* (sum of mid-chain monomethyl heptadecane homologous), where the dashed line marks a ratio of 1 (*i.e*., equal abundance of both biomarkers). In black, mean value of 𝛿^13^C of the different long-chain polyunsaturated alkanes that account for *Chloroflexus*-like microorganisms. We chose to represent the logarithm of the ratio to better visualized the differences between samples.

# References

Hayes J. M. (2001). Fractionation of the isotopes of carbon and hydrogen in biosynthetic processes. In *National Meeting of the Geological Society of America*, Boston, MA, November 2–4. DOI: 10.1515/9781501508745-006.

Jahnke, L. L., Eder, W., Huber, R., Hope, J. M., Hinrichs, K. U., Hayes, J. M., et al. (2001). Signature Lipids and Stable Carbon Isotope Analyses of Octopus Spring Hyperthermophilic Communities Compared with Those ofAquificales Representatives. *Applied and Environmental Microbiology* 67, 5179‑89.

Preuß, A., Schauder, R., Fuchs, G., and Stichler, W. (1989). Carbon Isotope Fractionation by Autotrophic Bacteria with Three Different C02 Fixation Pathways. *Zeitschrift Für Naturforschung C* 44, 397‑402. https://doi.org/10.1515/znc-1989-5-610.

Van der Meer, M. T. J., Schouten, S., and Sinninghe Damsté, J. S. (1998). The effect of the reversed tricarboxylic acid cycle on the ^13^C contents of bacterial lipids. *Organic Geochemistry* 28, 527-533.

Van der Meer, M. T. J., Schouten, S., de Leeuw, J. W., Ward, D., M. (2000). Autotrophy of Green Non-Sulphur Bacteria in Hot Spring Microbial Mats: Biological Explanations for Isotopically Heavy Organic Carbon in the Geological Record. *Environmental Microbiology* 2, 428‑35. https://doi.org/10.1046/j.1462-2920.2000.00124.x.

Van der Meer, M. T. J., Lammerts, L., Skirnisdottir, S., Sinninghe Damsté, J. S., and Schouten, S. (2008). Distribution and isotopic composition of bacterial lipid biomarkers in microbial mats from a sulfidic icelandic hot spring. *Organic Geochemistry* 39, 1015‑19. https://doi.org/10.1016/j.orggeochem.2008.04.002.

Van der Meer, M. T. J., Klatt, C. G., Wood, J., Bryant, D. A., Bateson, M. M., Lammerts, L. et al. (2010). Cultivation and genomic, nutritional, and lipid biomarker characterization of Roseiflexus strains closely related to predominant in situ populations inhabiting Yellowstone hot spring microbial mats. *Journal of Bacteriology* 192, 3033‑42. https://doi.org/10.1128/JB.01610-09.

Wilson, M. J. (2014). The structure of opal-CT revisited. *Journal of Non-Crystalline Solids* 405, 68‑75. https://doi.org/10.1016/j.jnoncrysol.2014.08.052.
